# *Vibrio* are a potential source of novel colistin-resistance genes in European coastal environments

**DOI:** 10.1093/ismeco/ycaf055

**Published:** 2025-05-05

**Authors:** Jamal Saad, Viviane Boulo, David Goudenège, Coralie Broquard, Karl B Andree, Manon Auguste, Bruno Petton, Yannick Labreuche, Pablo Tris, Dolors Furones, Augusti Gil, Luigi Vezzulli, Gianluca Corno, Andrea Di Cesare, Hugo Koechlin, Emilie Labadie-Lafforgue, Gaelle Courtay, Océane Romatif, Juliette Pouzadoux, Jean-Michel Escoubas, Dominique Munaron, Guillaume M Charrière, Eve Toulza, Marie-Agnès Travers, Caroline Montagnani, K Mathias Wegner, Delphine Destoumieux-Garzón

**Affiliations:** IHPE, Université de Montpellier, CNRS, IFREMER, Université de Perpignan Via Domitia, Place Eugène Bataillon, Montpellier 34090, France; IHPE, Université de Montpellier, CNRS, IFREMER, Université de Perpignan Via Domitia, Place Eugène Bataillon, Montpellier 34090, France; IFREMER, Infrastructures de Recherche et Systèmes d’Informations, Service Bioinformatique de l’Ifremer (SeBiMER)—Marine Bioinformatics Platform, Plouzané 29280, France; Alfred Wegener Institute (AWI)—Helmholtz Centre for Polar and Marine Research, Coastal Ecology, Waddensea Station Sylt, Hafenstrasse 43, 25992 List, Germany; Institut de Recerca i Tecnologia Agroalimentàries (IRTA), Aquaculture Program, Centre de La Ràpita, Crta. Poble Nou, km 5.5, 43540 La Ràpita, Spain; Department of Earth, Environmental, and Life Sciences, University of Genoa, Corso Europa 26, Genoa 16132, Italy; Laboratoire des sciences de l’Environnement MARin (LEMAR), Univ Brest, IFREMER, CNRS, IRD, Rue Dumont D’urville Plouzané 29280, France; IFREMER, Unité Physiologie Fonctionnelle des Organismes Marins, ZI de la Pointe du Diable, 29280 Plouzané, France; IHPE, Université de Montpellier, CNRS, IFREMER, Université de Perpignan Via Domitia, Place Eugène Bataillon, Montpellier 34090, France; IHPE, Université de Montpellier, CNRS, IFREMER, Université de Perpignan Via Domitia, Place Eugène Bataillon, Montpellier 34090, France; Institut de Recerca i Tecnologia Agroalimentàries (IRTA), Aquaculture Program, Centre de La Ràpita, Crta. Poble Nou, km 5.5, 43540 La Ràpita, Spain; Institut de Recerca i Tecnologia Agroalimentàries (IRTA), Aquaculture Program, Centre de La Ràpita, Crta. Poble Nou, km 5.5, 43540 La Ràpita, Spain; Department of Earth, Environmental, and Life Sciences, University of Genoa, Corso Europa 26, Genoa 16132, Italy; MEG Molecular Ecology Group—Water Research Institute, National Research Council of Italy, Largo Tonillo 50, 28922 Verbania, Italy; MEG Molecular Ecology Group—Water Research Institute, National Research Council of Italy, Largo Tonillo 50, 28922 Verbania, Italy; Laboratoire des sciences de l’Environnement MARin (LEMAR), Univ Brest, IFREMER, CNRS, IRD, Rue Dumont D’urville Plouzané 29280, France; IFREMER, Unité Physiologie Fonctionnelle des Organismes Marins, ZI de la Pointe du Diable, 29280 Plouzané, France; IHPE, Université de Montpellier, CNRS, IFREMER, Université de Perpignan Via Domitia, Place Eugène Bataillon, Montpellier 34090, France; IHPE, Université de Montpellier, CNRS, IFREMER, Université de Perpignan Via Domitia, Place Eugène Bataillon, Montpellier 34090, France; IHPE, Université de Montpellier, CNRS, IFREMER, Université de Perpignan Via Domitia, Place Eugène Bataillon, Montpellier 34090, France; IHPE, Université de Montpellier, CNRS, IFREMER, Université de Perpignan Via Domitia, Place Eugène Bataillon, Montpellier 34090, France; IHPE, Université de Montpellier, CNRS, IFREMER, Université de Perpignan Via Domitia, Place Eugène Bataillon, Montpellier 34090, France; MARBEC, Université de Montpellier, CNRS, IFREMER, IRD, 87 Av. Jean Monnet, 34200 Sète, France; IHPE, Université de Montpellier, CNRS, IFREMER, Université de Perpignan Via Domitia, Place Eugène Bataillon, Montpellier 34090, France; IHPE, Université de Montpellier, CNRS, IFREMER, Université de Perpignan Via Domitia, Place Eugène Bataillon, Montpellier 34090, France; IHPE, Université de Montpellier, CNRS, IFREMER, Université de Perpignan Via Domitia, Place Eugène Bataillon, Montpellier 34090, France; IHPE, Université de Montpellier, CNRS, IFREMER, Université de Perpignan Via Domitia, Place Eugène Bataillon, Montpellier 34090, France; Alfred Wegener Institute (AWI)—Helmholtz Centre for Polar and Marine Research, Coastal Ecology, Waddensea Station Sylt, Hafenstrasse 43, 25992 List, Germany; IHPE, Université de Montpellier, CNRS, IFREMER, Université de Perpignan Via Domitia, Place Eugène Bataillon, Montpellier 34090, France

**Keywords:** ocean, shellfish, bacteria, antibiotic, polymyxin, resistance, Vibrionaceae

## Abstract

Colistin is a widespread last resort antibiotic for treatment of multidrug-resistant bacteria. The recent worldwide emergence of colistin resistance (Col-R) conferred by *mcr*-1 in human pathogens has raised concern, but the putative sources and reservoirs of novel *mcr* genes in the marine environment remain underexplored. We observed a high prevalence of Col-R, particularly in *Vibrio* isolated from European coastal waters by using the same cohorts of oysters as bioaccumulators in three sites across Europe. The high sequence diversity found in the *mcr/ept*A gene family was geographically structured, particularly for three novel *eptA* gene variants, which were restricted to the Mediterranean (France, Spain) and occurred as a *dgk*A-*ept*A operon. The RstA/RstB two component system was shown to control both the *dgk*A-*ept*A operon and the Col-R phenotype. The analysis of 29 427 *Vibrionaceae* genomes revealed that this mechanism of intrinsic resistance is prevalent and specific to the Harveyi clade, which includes the human pathogens *Vibrio parahaemolyticus* and *Vibrio alginolyticus*. The operon conferred colistin-resistance when transferred to sensitive non-*Vibrio* strains. In general, *ept*A gene variants are widespread and evolved with the *Vibrio* lineage. They occur in clade-specific genomic environments, suggesting that *eptA* expression responds to distinct environmental signals across the *Vibrio* phylogeny. However, we also identified mobile *ept*A paralogues that have been recently transferred between and within *Vibrio* clades. This highlights *Vibrio* as a potential source of Col-R mechanisms, emphasizing the need for enhanced surveillance to prevent colistin-resistant infections in coastal areas.

## Introduction

The excessive and inappropriate use of antibiotics in human and veterinary medicine has led to the spread of antimicrobial resistance genes (ARGs) by creating selective pressures favoring the development of resistant bacteria [1]. If this problem is not addressed, it is estimated that by 2050, antimicrobial resistant bacteria (ARB) could lead to an annual loss of ~10 million lives and limit options for effectively treating bacterial infections [2].

The antibiotic properties of cationic cyclic antimicrobial peptides belonging to the group of polymyxins (e.g. colistin, polymyxin B) is well known, but the use of these peptides for human therapy is limited due to strong side effects. For this reason, limited resistance is reported in clinical settings, promoting the use of polymyxins as a last resort antibiotic for treatments of multidrug-resistant infections [3–5]. Preservation of long-term effectiveness of polymyxins is thus of primary importance for human health. Regrettably, the use of colistin has increased as a growth promoter in poultry and swine farms worldwide. This has resulted in the rapid spread of colistin resistance in Gram-negative bacteria that are clinically significant on a global scale [6, 7]. The global spread of polymyxin-resistant bacteria in clinical and environmental settings has become a major concern in the treatment of multidrug-resistant pathogens in recent years [8].

Polymyxins bind to the negatively charged Lipid A component of lipopolysaccharides at the outer membrane of Gram-negative bacteria [5, 9], then they disrupt the structure of the outer membrane, leading to the increase of cell permeability and subsequent cell death [10]. Resistance to polymyxins by Gram-negative bacteria can rely on different mechanisms all leading to the reduction of Lipid A negative charges thus decreasing electrostatic interactions with polymyxins [11]. In Gram-negative bacteria, including in human and animal pathogens, colistin resistance is frequently chromosomally encoded by *ept*A and *pmr*HFIJFKLM (also referred to as *arn*BCADTEF) [12, 13]. These genes catalyze the addition of phosphoethanolamine (PEtN), or the addition of a 4-amino-4-deoxy-L-arabinose (L-Ara4N) to the phosphate groups of lipid A moieties [14]. In various bacterial genera the activation of the *ept*A and *arn*BCADTEF expression is controlled by the PmrA/B and/or PhoP/Q two-component systems (TCS; for review, see [15]). Additionally, a plasmid-mediated mechanism of resistance to colistin was discovered <10 years ago and involves *mcr*-1 (for mobile colistin resistance) [6]. It is a rare example of a recent ARG capture and spread. The emergence and rapid spread of *mcr-*1 among various Gram-negative bacteria has alerted health organizations worldwide (Europe, Asia, North America, and Africa) [16], and the number of newly reported *mcr* genes is ever-growing since 2015 [17]. Furthermore, recent research indicates that the majority of mobile colistin resistance gene variants (*mcr*1–9) have originated from environmental bacteria, particularly from aquatic sources [18]. Similar to *eptA, mcr-*1 encodes a phosphoethanolamine transferase (PET) but its expression is not dependent on a regulatory system [11].

The rapid spread of colistin resistance from environmental sources highlights the urgent need to enhance our understanding of the prevalence and distribution of colistin-resistant bacteria and their associated resistance genes in aquatic ecosystems. This calls for research that emphasizes the interconnectedness of humans, animals, and the environment (i.e. the One-Health concept [19, 20]). However, the marine environment remains largely unexplored regarding antimicrobial resistance (AMR), especially in Europe. Studying AMR in coastal marine environments is particularly important because: (i) coastal systems are highly exposed to human contaminants that may select or co-select for ARGs; (ii) coastal systems are highly interconnected through international trade, which favors the worldwide spread of ARGs; (iii) human populations live along coasts and depend on marine environments as a food source [21], which increases the risk of transmission; and (iv) coastal waters and sediments, wastewater discharge and marine aquaculture can act as sources/reservoirs of ARGs. All of the above have contributed to localized increases in the abundance of ARGs [22, 23]. Still, the study of AMR in marine waters lags behind in comparison to other environments, and there is a lack of understanding of the role these environments play in the global cycle of AMR.

The marine environment may harbor diverse ARGs, flourishing under human-induced pressures. Almost all known variants of the *mcr* gene that have been reported thus far are from aquatic bacteria from diverse environments [18]. Among them, aquatic bacteria from the *Shewanella* genus are considered as a source of *mcr-*4 [18]. Moreover, *mcr-*1 was found recently in colistin-resistant bacteria in marine coastal waters from Norway and Croatia, as well as in aquaculture ponds from South China, which may constitute reservoirs [24–26]. Some recent studies have also shown the role of variants of the *ept*A gene in colistin resistance in strains of *Vibrio cholerae* [27]*, Vibrio parahaemolyticus* [28] *Vibrio vulnificus* [29] and *Vibrio fisheri* (*Aliivibrio fisheri*) [30]. Given the limited data available on the frequency and distribution of colistin-resistant bacteria in marine environments, along with the evidence of colistin-resistant *Vibrio* species with pathogenic potential in humans and marine fauna [31], there is an urgent need to increase our understanding of the medical and ecological significance of this phenomenon.

To further evaluate the potential role of the coastal environment in the emergence and circulation of colistin resistance genes, we determined the prevalence of *mcr*/*ept*A genes in culturable marine bacteria in coastal waters across Europe, as well as in *Vibrionaceae* in general. We targeted three different European areas used for oyster farming that are impacted by anthropogenic pollution from tourism and/or agriculture: Sylt (Germany), the Ebro Delta (Spain), and Thau Lagoon (France). We characterized the prevalence, diversity, and distribution of *mcr/ept*A by using the same cohorts of oysters as bioaccumulators that were incubated in all sites for tracking AMR, as they concentrate bacteria from the environment by filter feeding [32]. Our data revealed an unexpected diversity of *ept*A in *Vibrio*, with a clearly structured geographic distribution of *ept*A variants across Europe. Functional genetic experiments were used to demonstrate the mechanisms of colistin resistance conferred by the newly discovered *eptA* gene variants from the *Vibrio Harveyi* clade. The discovery of highly diverse and prevalent mechanisms of colistin resistance as well as evidence of recent mobilization in *Vibrionaceae* highlights the underestimated risk of the emergence of colistin resistance in European coastal environments and warrants further investigation.

## Materials and methods

### Animals

Specific-pathogen free (SPF) diploid oysters were produced during Spring 2021 from 166 wild genitors at the Ifremer hatchery of Argenton (PHYTNESS Ifremer research unit, France), as described previously [33, 34]. Oysters were transferred after 6 weeks to the Ifremer nursery of Bouin (EMMA Ifremer research unit, France), where they were maintained under controlled biosecured conditions with filtered and UV-treated seawater enriched in phytoplankton (*Skeletonema costatum*, *Isochrysis galbana*, and *Tetraselmis suecica*). Before transfer to the field, the SPF status of the animals was confirmed by (i) the absence of OsHV-1 DNA detection by quantitative real-time Polymerase Chain reaction (qPCR), (ii) a low *Vibrio* load (~10 CFU/mg of oyster tissue) determined by isolation on selective Thiosulfate Citrate Bile Salts Sucrose medium (TCBS agar). The absence of World Organisation for Animal Health (WOAH) listed parasites (*Bonamia* sp., *Marteilia* sp., *Perkinsus* sp., *Mikrocytos* sp. and *Haplosporidium* sp.) was confirmed on histological sections of 114 oysters by an independent laboratory (LABOCEA, France). Oysters were observed to remain free of any abnormal mortality until use.

### Study area, sample collection, and microbial isolation

**Figure 1:**
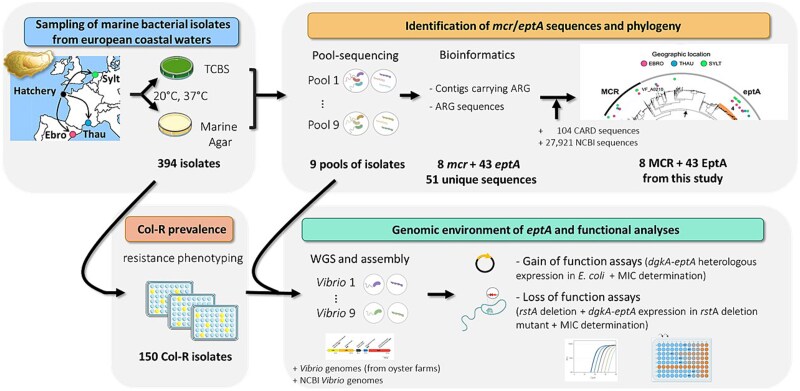
Overview of the sampling and screening for mechanisms of colistin resistance diploid oysters produced during spring 2021 in a French hatchery were immersed at the juvenile stage (6 months old) in three different European locations used for oyster culture: Sylt (Germany), Thau lagoon (France), and Ebro delta (Spain). After being exposed during 2–3 weeks in the environment, 60 oysters were collected at each sampling site and homogenized for CFU isolation. In total, 394 bacterial isolates growing on TCBS or marine agar at 20°C or 37°C were sampled. Pool-sequencing of bacterial DNA was performed according to conditions of isolation (site, temperature and culture medium, see Table S1 for pool sequencing metadata). To make an inventory of colistin resistance genes, the 9 pools of bacterial DNA were sequenced. After classical pre-processing steps, reads were assembled into contigs that were screened for ARG (see Fig. S1 for the bioinformatics pipeline). A total of 8 *mcr* and 43 *ept*A unique sequences were identified (see Table S2 for origin, Genbank ID, taxonomic affiliation, and normalized read count of each sequence). Sequences were classified as *mcr* or *ept*A through a large phylogeny including 104 curated references from the CARD database and 27 921 sequences deposited in the NCBI (see Table S3 for sequence identity between newly identified members of the *ept*A gene family). In parallel, the 394 isolates were screened for resistance to 5 μg/ml colistin in Zobell medium (see Dataset 1 for phenotypes). A total of 150 col-R isolates were identified, with different abundance according to geographic sites (Fig. S2). 9 genomes of col-R isolates randomly chosen in *ept*A-positive pools were sequenced. Genomic organization of the *eptA* locus allowed the description of a conserved genomic environment of *eptA* in *vibrio* of the Harveyi clade (Table S2). Finally, the role of these conserved genes in colistin resistance was demonstrated through gain of function and loss of function assays.

The same SPF oyster batches (i.e. oysters with identical life history traits) were deployed at the juvenile stage (6 months old) in three different European locations used for oyster culture: SYLT in Germany (N 55° 1′ 42.539′, E 8° 26′ 1.953′), THAU lagoon (N 43°26.058′, E 003°39.878′) in France, and EBRO delta (N 40°37.106880′, E: 0°37.318320′) in Spain (Fig. 1). Two to three weeks exposures in the environment were performed at the end of the year 2021 (THAU: Bouzigues, from 4 October 2021 to 18 October 2021, EBRO: Alfacs bay, from 8 October 2021 to 25 October 2021, and SYLT: Königshafen, from 22 December 2021 to 10 January 2022). No oyster mortalities were recorded during the field exposures. After being exposed to their respective environments, 60 oysters were collected at each sampling site. Tissues were homogenized in artificial seawater (ASW: 400 mM NaCl, 20mM KCl, 5 mM MgSO4, 2 mM CaCl2) using an ultra-Turrax apparatus [35]. The homogenates were pooled and plated in replicates on TCBS agar (Difco™) agar plates for *Vibrio* isolation and on marine agar (Difco™) as a non-selective agar for marine bacteria. Duplicate plates were incubated at 20°C or 37°C. Bacteria were isolated after 24–48 h (Table S1). After colony purification on Zobell medium (ASW supplemented with 0.4% bactopeptone and 10% yeast extract, pH 7.8), a glycerol stock of each isolate was conserved at −80°C. Up to 48 isolates were conserved per condition of isolation (TCBS 20°C, marine agar 20°C, TCBS 37°C, marine agar 37°C).

### Deoxyribonucleic acid extraction, pool-sequencing, and whole genome sequencing

For pool-sequencing, the 394 bacterial colonies isolated from oyster flesh were cultured overnight in 96 well microplates in liquid Zobell medium at either 20°C or 37°C, according to their conditions of isolation. After OD_600_ reading, bacterial suspensions were pooled in equal amounts before DNA extraction. Pools corresponding to 18 to 92 colonies were created based on the geographic origin, culture medium and temperature of isolation (Table S1). DNA was extracted with the NucleoSpin Tissue kit for DNA from cells and tissue (Macherey-Nagel). For single genome sequencing, bacterial colonies were cultured overnight in individual sterile polypropylene tubes and DNA was extracted from cultures with the MagAttract HMW DNA Kit (Qiagen, France). The extracted DNA was quantified using a Qubit High Sensitivity Assay Kit (Life Technologies, Carlsbad, USA), and sequencing was carried out at the Bio-Environment platform (University of Perpignan Via Domitia) using the Nextera XT DNA Library Prep Kit (Illumina) according to the manufacturer’s instructions, with 1 ng of DNA. The quality of the libraries was checked using a High Sensitivity DNA chip (Agilent) on a Bioanalyzer. Sequencing was performed on a NextSeq 550 instrument (Illumina) in 2 × 150 paired-end mode, resulting in an average mean reads of 45 000 000 bp for pool sequencing, and 243 Mb for whole genomes (mean coverage 47X).

### Bioinformatics analysis tools

#### Pool-sequencing

FastQC was used to check the quality of reads, followed by trimming using Trimmomatic V-0.38[36] to trim leading/trailing bases with quality scores below 30. The recovered reads were assembled into contigs using MEGAHIT V-1.2.9 [37]. The Meta-marc tool (database model 3) was then used to identify the contigs carrying the *eptA/mcr* resistance genes (Fig. S1). The contigs recovered carrying the *eptA/mcr* resistance genes were annotated using Prokka and predicted coding sequences were specifically re-analyzed for colistin resistance genes using Meta-marc. Sequences annotated as *ept*A and *mcr* according to Meta-marc and Prokka, were validated by BlastP against the NCBI and the CARD database V-3.2.7. Subsequently, we eliminated incomplete *ept*A and *mcr* sequences (i.e. partial sequences missing a 5′ and/or 3′ region) from our analysis. Full length gene sequences were translated *in silico* and the resulting amino acid sequences were aligned by MAFFT V-7.407 (https://ngphylogeny.fr/tools/). A phylogenetic tree was generated through maximum likelihood analysis of deduced MCR/EptA amino acid sequences using PhyML V-3.0 (https://ngphylogeny.fr/tools/) with the WAG model and 100 bootstrap replicates. Pool-sequencing raw data and complete *eptA* gene sequences were deposited on GenBank under accession numbers SAMN37810832 to SAMN37810840 and OR578979 to OR579029, respectively. MCR/EptA sequences from the pool sequencing were assigned to a bacterial genus based on their sequence similarity to known NCBI sequences (>90% sequence identity over the full-length sequence). Genus assignment was then confirmed through their clustering within the *Vibrionaceae* phylogenetic tree (29 244 genomes assemblies, see below).

#### Whole genome sequencing of single bacterial isolates

A total of nine *Vibrio* genomes were sequenced. The quality assessment and reads trimming steps were performed as described for pool-sequencing. The obtained reads were then assembled into contigs using Spades V-3.15.4 within the Galaxy Europe platform [38] (see assembly quality in Table S4). Default parameters were used (“Isolate”, “Automatic k-mer selection”, Phred quality offset adjustment, and coverage cutoff in the assembly of individual bacterial genomes). In order to detect the presence of the *ept*A or *mcr* genes, the assembled genomes were annotated in MAGe (https://mage.genoscope.cns.fr/). When feasible, a taxonomic affiliation was assigned to the selected isolates at the species level using Average Nucleotide Identity (ANI) and DNA:DNA hybridization (dDDH) percentages. This analysis was conducted utilizing Defast (DDBJ Fast Annotation and Submission Tool) available at https://dfast.ddbj.nig.ac.jp/, in conjunction with genome clustering tools through MAGe and the reference strain genomes obtained from Type Strain Genome Server (TYGS; https://tygs.dsmz.de/). Raw reads and genome assemblies were deposited on ENA under project number PRJEB67316.

#### Genetic diversity of detected *ept*A and *mcr* genes, and their genomic environment

In order to investigate the diversity of the 51 detected *mcr/ept*A sequences, we conducted a two-step analysis. First, to assign them to the *mcr/ept*A ontology, we compared the amino acid sequences encoded by these genes to 104 annotated MCR/EptA sequences from the CARD reference database. Subsequently, to extend our analysis to the entire *Vibrionaceae* family, we used known *mcr/ept*A sequences from the CARD database and the 51 sequences from the present study to screen for homologous sequences in a collection of 29 244 *Vibrionaceae* assemblies (GenBank, July 2023) using diamond BlastP (v2.1.9.163) [39] with 30% identity and 50% coverage as selection criteria. Alignment of multiple protein sequences was performed using FAMSA (v2.2.2) [40] and trimmed using trimAI with gappyout method (v1.5) [41]. A phylogenetic tree was constructed using FastTree with LG model (v2.1.11) [42] and visualization was done using iTOL [43].

Additionally, we examined the genomic environment of all *mcr/ept*A genes from the 29 244 *Vibrionaceae* assemblies. Neighbor genes from +4 to −4 were clustered with the diamond cluster module. After filtering out incomplete environments (i.e. those which lacked genes at +4 or − 4 due to contig breaks), the different genomic environments were positioned on a *Vibrionaceae* phylogenetic tree. For that, a multilocus sequence analysis of *Vibrionaceae* genomes was performed based on 8 genes recommended by *Vibrio* clade 3.0 [44], namely *fts*Z, *gap*A, *mre*B, *rpo*A, *top*A, *gyr*B, *pyr*H, and *rec*A. Alignment of the 8 concatenated nucleic acid sequences was done with halign (v3.0.0) [45]. A phylogenetic tree was constructed with FastTree with a GTR model. iTOL was used for visualization. ISfinder (https://isfinder.biotoul.fr/blast.php), PlasmidHunter [46] and MetaPhinder-2.1 (https://cge.food.dtu.dk/services/MetaPhinder/) were used to search the insertion sequences, mobile elements and bacteriophage sequences within a 25 000 bp average region surrounding the *ept*A gene.

#### Data normalization and statistical analysis

Bowtie2 V-2.5.1 [47] was employed with its default settings to align reads to the gene sequences of interest. This approach provides read counts for each identified sequence (Table S2). To normalize the number of reads of each identified sequence, the following formula was used: Normalized data = (mapped reads of each sequence/total assembly reads) × 10^6^.

Furthermore, we investigated the geographical structuring of molecular diversity in EptA and MCR sequences by analyzing the distance matrix underlying the amino acid sequences phylogeny (see above). This involved conducting pairwise comparisons of distances within and between sites using a permutational analysis of variance (PERMANOVA). The PERMANOVA was implemented in the R package *vegan*, utilizing the *adonis*2 function. (https://github.com/vegandevs/vegan). Initially, we conducted an analysis of variation using all sites collectively. Subsequently, we performed pairwise comparisons to detect low-level structuring.

### Colistin susceptibility testing

MICs of colistin were determined against *Vibrio* isolates and recombinant *Escherichia coli* strains constructed in the present study following the microdilution assay from the EUCAST V14.0 guidelines. Colistin sulfate (Thermo Fisher) corrected for activity units was tested in the range of 0.125 to 16 μg/ml, at 35°C, in cation-adjusted MHCA. Each well was seeded with 5.10^5^ CFU/ml. MIC values are expressed as the lowest colistin concentration tested that causes 100% of growth inhibition after a 18 h incubation. In parallel, we performed MIC determination in Zobell medium, which mimics the seawater composition. For quality control (QC), we used *Escherichia coli* O6 (ATCC 25922) with the colistin QC MIC range provided by EUCAST v14.0 (0.25–1 μg/ml). In the absence of colistin breakpoint for *Vibrio*, we used EUCAST breakpoints for *Enterobacterales* resistance in tables V14.0 (MIC >2 μg/ml).

For a rapid screening of Col-R on 139 bacterial isolates from the Thau lagoon, France, 184 from Ebro, Spain and 96 from Sylt, Germany and control *Vibrio* strains from previous studies (see Tables S4, Dataset 1), we used the same liquid broth inhibition assay with a fixed colistin concentration (5 μg/ml). Screening was performed in Zobell medium at 20°C or 37°C, according to the conditions of first isolation. Assays were performed in duplicate wells. Strains were considered resistant when duplicate wells grew in the presence of 5 μg/ml colistin (i.e. 2.5 fold the clinical breakpoint) after a 18 h incubation.

### Cloning *dgk*A and *ept*A genes

Gene variant *ept*A-1 and the operon *dgk*A-*ept*A-1 were amplified by PCR from colistin resistance strain *Vibrio owensii* Th15_Z_G08 (Table S4). The *ept*A-4 variant was amplified from *Vibrio splendidus* 7T7_2. Primer sets used for gene amplification are listed in Table S5. PCR were performed in a 25 μl total volume under the following conditions: initial denaturation at 95°C for 30 s, followed by 35 cycles of 95°C for 5 s, 69°C for 30 s, and 72°C for 30 s and by a final extension at 72°C for 2 min. Amplicons were cloned in the pBAD-TOPO expression vector (Invitrogen, France) under the control of the pBAD inducible promoter and transformed into *E. coli* TOP10 competent cells according to manufacturer’s instructions. Recombinant colonies were tested for the presence of specific *ept*A and *dgk*A genes by standard PCR and by whole plasmid sequencing using Oxford Nanopore Technologies sequencers (Eurofins, France).

### Heterologous expression of *dgk*A and *ept*A genes in *Escherichia coli*

Recombinant *E. coli* TOP10 carrying *ept*A-1, *dgkA*-*ept*A-1 or *ept*A-4 in the pBAD-TOPO vector were tested for colistin resistance. In brief, bacterial cells were cultured at 37°C in Luria-Bertani (LB) broth in the presence of 0.2% arabinose to induce the P_BAD_ promoter. Recombinant bacteria were considered colistin-resistant if they were able to grow in LB containing 5 μg/ml colistin in the microtiter plate assay described above.

### Generation of mutants in the regulation systems *rst*A/B

The *Vibrio* strain TH15_F5_F11 carrying *dgk*A-*ept*A-1 preceded by *rst*A/B (Col-R) was grown at 37°C in LB or LB-agar (LBA) + 0.5 M NaCl. *E. coli* strains were grown at 37°C in LB broth and on LB medium for cloning and conjugation experiments. Chloramphenicol (Cm, at 5 or 25 μg/ml for *Vibrio* and *E. coli,* respectively), thymidine (0.3 mM) and diaminopimelate (0.3 mM) were added as supplements when necessary. Induction of the P_BAD_ promoter was achieved by the addition of 0.2% L-arabinose to the growth medium and, conversely, was repressed by the addition of 1% D-glucose where indicated. All plasmids used or constructed in the present study are described in Table S4. Gene deletion was performed by allelic exchange using the pSW7848T suicide plasmid [48, 49]. To this end, two ≈500 bp fragments flanking the target gene were amplified (Table S5), cloned into pSW7848T as previously described [50], and transferred by conjugation from *E. coli* as donor to *Vibrio* as recipient. Subsequently, the first and second recombination’s leading to pSW7848T integration and elimination were selected on Cm/glucose and arabinose-containing media, respectively. Deletion mutants were screened by PCR using external primers flanking the target gene. For the complementation experiments, the gene was cloned into the stable pMRB plasmid, resulting in constitutive expression from a P_LAC_ promoter [51]. Conjugations between *E. coli* and *Vibrio* were performed at 37°C [48].

### Reverse transcription quantitative polymerase chain reaction

In this study, we employed the DirectZol RNA Miniprep kit (R2051) provided by ZymoResearch to extract total RNA from Trizol conserved samples obtained from both wild and mutant strains, following the manufacturer’s instructions. The extraction process was performed in duplicate for each condition at two different growth stages, specifically the exponential and stationary phases. To eliminate any genomic DNA contamination, the RNA was treated with DNase I. To determine the concentration of the total RNA, we used a NanoDrop spectrophotometer from ThermoFisher Scientific. The cDNA was produced using M-MLV Reverse Transcriptase M1302 (Sigma-Aldrich, France) with 1 μg of extracted RNA. Real-time quantitative PCR (qPCR) was performed at the MGX platform in Montpellier using SYBR Green I Master mix (Roche) and primers at 0.2 µM. The MGX platform employed the Light-Cycler 480 System from Roche. The primers used for qPCR are listed in Supporting Information (Table S5). To analyze the relative expression levels, we employed the 2^-ΔΔCq^ method developed by Pfaffl in 2001 [52]. For normalization, two genes [6PFK (VS_2913) and CcmC (VS_0852)] were chosen due to their constitutive expression across various conditions in both the RNAseq and reverse transcription quantitative PCR (RT-qPCR) analyses [53]. For the study herein, we designed and validated specific primers for these genes on *Vibrio harveyi* Th15_F5_F11 (Table S5).

## Results

### Colistin-resistant bacteria are abundant in oysters in European coastal environments

To estimate the prevalence of colistin resistance (Col-R) in culturable marine bacteria from European coastal ecosystems, we immersed Specific Pathogen Free (SPF)-oysters in three sites either containing natural beds (Sylt, Germany), or oyster farms (Ebro, Spain and Thau, France). After 2–3 weeks, marine bacteria isolated from the SPF-oysters, on either marine agar or TCBS agar at 20°C or 37°C, were tested for Col-R using a microtiter plate assay (Fig. 1). Using Zobell medium, which mimics the composition of seawater, the minimal inhibitory concentration (MIC) of >5 μg/ml for colistin was used to identify Col-R strains (Table 1). Bacteria isolated on marine agar showed frequent resistance to colistin. Out of 87 bacterial isolates from Thau lagoon (France), 28 (32.1%) were Col-R. Most of these Col-R isolates (27/28) were isolated at 37°C. In the Ebro delta (Spain), 17/140 isolates were Col-R (12.1%) and most of them (11/17) were isolated at 37°C. In Sylt (Germany), 37/51 isolates were Col-R (72.5%), and most of them (23/37) were isolated at 37°C (Table 1). Col-R phenotypes were even more prevalent among bacteria isolated on TCBS (selective for *Vibrio*). Out of the 52 isolates obtained on TCBS media from oysters collected in Thau lagoon, 16 (30.8%) were Col-R. Among them, 11/16 were isolated at 20°C and 5/16 were isolated at 37°C. In Ebro, 10 out of 50 isolates (20%) were Col-R. Among them, 5/10 were isolated at 20°C and 5/10 were isolated at 37°C. In Sylt, most isolates (41/45; 91.1%) were Col-R; all of them were isolated at 20°C (no bacterial isolates at 37°C; Table 1). Overall, our sampling highlighted a remarkable prevalence of colistin resistant isolates in culturable bacteria accumulating in oysters immersed in European coastal waters, particularly in Northern Germany (general linear model, *P =* 1.5 × 10^−5^). In France and Spain, the percentage was particularly high in bacteria isolated at 37°C (*P =* 3.8 × 10^−5^). There was also a clear effect of the isolation on TCBS medium (*P =* 7.7 × 10^−3^), suggesting higher Col-R prevalence in *Vibrio* (Fig. S2).

**Table 1:** Colistin-resistant (Col-R^*^) bacterial isolates^**^ across European oyster farms.

Sampling site	Isolation medium	No. isolates (20°C; 37°C)	No. Col-R isolates (20°C; 37°C)	% Col-R isolates
THAU	Marine agar	87 (41; 46)	28 (1; 27)	32.1
EBRO	Marine agar	140 (96; 44)	17 (6;11)	12.1
SYLT	Marine agar	51 (19;32)	37 (14;23)	72.5
THAU	TCBS	52 (47; 5)	16 (11; 5)	30.8
EBRO	TCBS	50 (28; 22)	10 (5; 5)	20.0
SYLT	TBCS	45 (45; 0)	41 (41; 0)	91.1

^*^Col-R, MIC >5 μg/ml in Zobell medium.

^**^Bacteria isolated on TCBS or Marine agar medium.

### High diversity of *mcr*/*ept*A genes in oysters in European coastal systems

We used pool-sequencing to capture the diversity of *mcr*/*ept*A resistance genes circulating in culturable bacteria from European marine coastal systems. Pool-sequencing was performed on a total of 394 bacterial isolates pooled by site and isolation conditions (culture medium, temperature) with an average number of 4 × 10^7^ reads per pool (99 to 149 strains per sampling site; see Table S1). A total of 51 complete unique nucleotide sequences related to *mcr*/*ept*A were found in the contigs from the pool-sequencing after Prokka annotations (Fig. 1). Among them, 31 and 22 sequences were carried by bacteria isolated on either marine agar or TCBS, respectively (Table S1). The diversity of the MCR/EptA amino acid sequences was studied by reconstructing their molecular phylogeny. Amino acid sequences deduced from the pool sequencing were compared to the 104 Mcr-1 to −10 amino acid sequences present in the CARD database (Fig. 1). The whole set of sequences was also used to identify MCR/EptA sequences encoded in the 29 427 *Vibrionaceae* genome assemblies found in GenBank. A total of 27921/29427 genomes encoded at least one MCR/EptA, representing 4075 distinct amino acid sequences among 31 813 protein hits, which were included in the analysis (Fig. 1).

**Figure 2:**
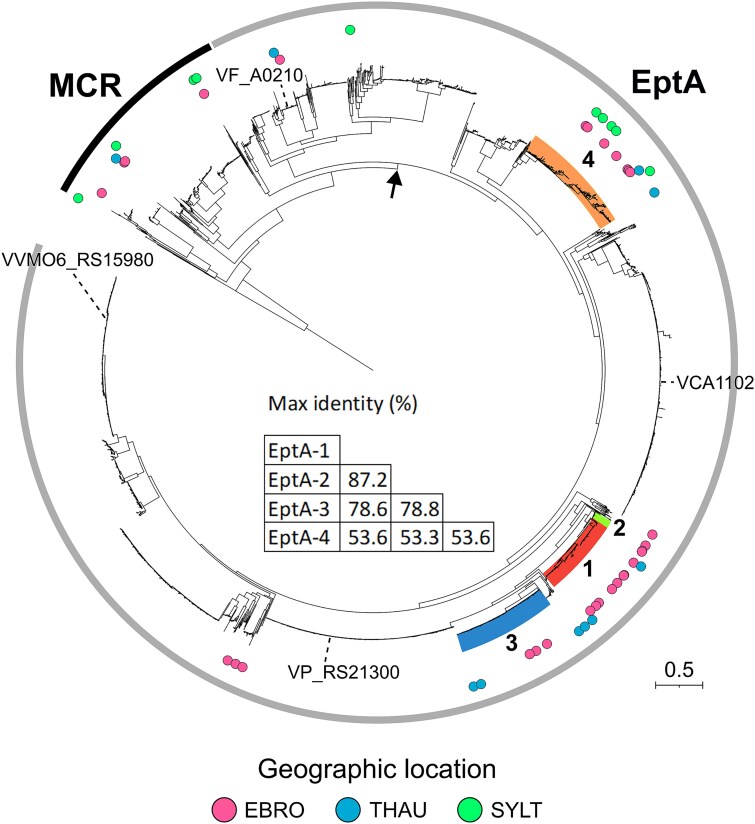
Clustered distribution of *mcr/eptA* gene variants across European coastal environments. The 51 detected EptA and MCR variants from the present study are included in a phylogenetic tree (colored circles) together with 4075 distinct MCR/EptA sequences found in 27 921 *Vibrionaceae* assemblies carrying *mcr/ept*A genes. Numbers in boldface refer to the EptA-1, EptA-2, EptA-3 and EptA-4 variants described in the present study. Maximum identity between newly identified EptA protein variants is displayed. The geographic origin of each sequence (Ebro, Sylt, Thau) is represented in by colored circles. Sequences used as reference are MCR-1 to 10 sequences found in the CARD database as well as 4 EptA sequences from functionally characterized EptA variants (VP_RS21300, VVMO_RS15980, VCA1102 and VF_A0210). A phylogenetic tree generated from deduced EptA/MCR amino acid sequences was constructed using FastTree with LG model and visualization was done using iTOL. Corresponding protein sequences were obtained from the pool-sequencing of bacteria isolated both on marine agar and TCBS medium in three European regions. See Fig. S3 for an illustration including conditions of isolation and read counts. The node that separates MCR (outer black arc) from EptA sequences (outer grey arc) is indicated by an arrow.

EptA and MCR sequences could clearly be distinguished based on the molecular phylogeny of the 4075 deduced amino acid sequences (Fig. 2). The EptA clade gathered a highly diversified set of sequences from *Vibrionaceae*, including the four previously characterized EptA variants from *V. cholerae* [27]*, V. parahaemolyticus* [28]*, V. vulnificus* [29] and *Varicus fisheri* [30] (Fig. 2). Most of the sequences from the pool-sequencing (43/51) were assigned to EptA whereas only 8/51 sequences were assigned to MCR (Fig. 2). The 51 sequences from this study were only found in the family *Vibrionaceae.* The EptA sequences were found in the genera *Vibrio*, *Photobacterium,* and *Allivibrio,* whereas the MCR sequences were found in the genera *Vibrio, Photobacterium, Shewanella,* and *Pseudoalteromonas* (Fig. S3, Table S2). This taxonomic assignment was based on protein sequence similarity using 90% amino acid sequence identity over their full-length sequence as a threshold for genus assignment based on taxonomy of NCBI sequences. Most MCR variants were previously unreported and the majority clustered with MCR-4 (Fig. S3). Compared to EptA variants, MCR was far less abundant, both in terms of sequence diversity and read counts (Fig. 2, Fig. S3).

### EptA variants specific of the Harveyi and Splendidus clades are dominant in oysters

To gain insight into the origin and diversity of *ept*A genes circulating in European coastal environments, we analyzed the 51 *eptA/mcr* sequences found in oyster samples from Thau, Ebro and Sylt in terms of sequence polymorphism, genomic environment and assignment to *Vibrionaceae* clades and species. Four predicted EptA proteins (EptA-1 to -4) encoded by unique nucleotide sequences were dominant in oysters, both in terms of sequence diversity (Fig. 2) and read counts (Fig. S3). EptA-1 to -4 sequences harbored the catalytic threonine conserved in EptA orthologs functionally characterized in *Vibrionaceae* (Fig. S4) as well as in MCR/EptA proteins from *Enterobacteriaceae* [27]. EptA-1 to -3 clustered separately from EptA-4 in the MCR/EptA phylogeny (Fig. 2). Compared to previously characterized EptA variants, EptA-1 to -3 amino acid sequences showed a maximum identity of 80.9%–83.6% with VP_RS21300 from *V. parahaemolyticus* and 69.9%–72.9% with VVMO6_RS15980 from *V. vulnificus*. Only 58.2%–58.7% maximum identity was found with VCA1102 from *V. cholerae* and 42.5%–44.5% with VF_A0210 from *V. fisheri*. EptA-4 was far more divergent with only 43.6%, 55.2%, 54.6% and 56.6% maximum identity with VF_A0210, VCA1102, VP_RS21300 and VVMO6_RS15980 respectively (Table S3).

**Figure 3:**
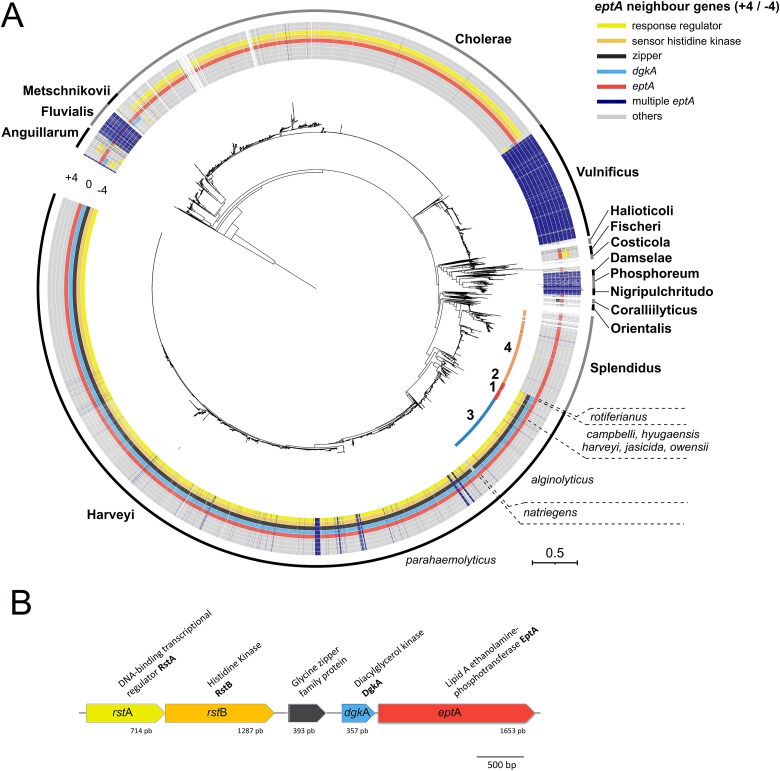
The genomic environment of *ept*A gene variants has evolved with the phylogeny of *Vibrionaceae*. (A) Distribution of *mcr/ept*A genomic environments (−4/+4 genes) along a multi-locus sequence analysis (MLSA) of *Vibrionaceae*. The *Vibrionaceae* phylogenetic tree was constructed from 23 642 genome assemblies with sufficient quality to be included in a MLSA (Dataset 2). The MLSA was based on 8 polymorphic genes *fts*Z, *gap*A, *mre*B, *rpo*A, *top*A, *gyr*B, *pyr*H, *rec*A according to *Vibrio* clade 3.0 [44]. Clades and species of *Vibrio* are indicated in boldface and italics, respectively. Numbers on the inner circle indicate the phylogenetic positioning of EptA-1, EptA-2, EptA-3, and EptA-4 variants identified in the present study (Table S2). Outer circles show genes in the genomic environment of *mcr/eptA* genes with the following color code: response regulator (RR), sensor histidine kinase (HK), *dgk*A, glycine zipper family protein. Note that in the *Cholerae* clade, the HK and RR genes are located at positions +1 and + 2 relative to *eptA* and are transcribed in the opposite direction of *eptA*. When >1 *mcr/ept*A gene copy were found in *vibrio* genomes, their genomic environments were not displayed (dark blue). (B) Newly discovered genetic environment in the Harveyi clade. A *rst*A*/rst*B (response regulator/histidine kinase) two component system is located upstream *dkgA* and *eptA*. A gene encoding a glycine zipper family protein separates *rst*A*-rst*B from *dgk*A*-ept*A. The figure is based on an *ept*A-1 sequence from *V. harveyi*.

Genes encoding EptA-1 to -3 shared a previously undescribed genomic environment specific to the Harveyi clade, when considering the four genes upstream and downstream of the *mcr/eptA* sequences in a *Vibrionaceae* phylogenetic tree (Fig. 3A). This Harveyi-specific genomic environment consists of five conserved genes: *rst*A-*rst*B-Glycine zipper family protein-*dgk*A-*ept*A (Fig. 3B). Remarkably, within this genomic environment, EptA polymorphisms appears to have followed differentiation between *Vibrio* species. Indeed, EptA-1 was found in the species *V. harveyi, V. campbelli, V. jasicida, V. owensii,* and *V. hyugaensis;* EptA-2 was found in *Vibrio rotiferianus*; and EptA-3 was found in *Vibrio alginolyticus,* resembling the phylogeny of the *Vibrionaceae* (Fig. 3A). We also found a very large cluster of genes encoding EptA sequences similar to VP_RS21300 (reference sequence not sampled in our pool sequencing) near EptA-1 to −3 on the MCR/EptA phylogenetic tree (Fig. 2). They were ubiquitous in the NCBI database for *V. parahaemolyticus,* as shown in the *Vibrionaceae* phylogeny (Fig. 3). Finally, an additional EptA sequence from our pool sequencing, clustered close to EptA-3 (Fig. 2). It matched with *V. alfacsiensis* (>90% amino acid sequence identity over its full-length sequence compared to sequences found in NCBI)*,* also belonging to the Harveyi clade (Table S2, Dataset 2).

The gene encoding EptA-4, also abundant in our pool sequencing (Fig. 2) occurred in a completely different genomic environment, lacking both *dgk*A and the *rstA/rstB* two-component regulatory system (Fig. 3). Within the *Vibrionaceae* phylogeny EptA-4 was specific to the Splendidus clade (Fig. 3A).

Finally, two EptA sequences almost identical to *V. fisheri* VF_A0210 (>98.7% identity) were carried by bacterial isolates from our European sampling and clustered apart from the newly discovered EptA-1 to −4 protein variants (Fig. 2). The remaining sequences did not share any significant conservation of the sequence/synteny with other EptA variants from our study and were present in sequences of *Vibrio, Allivibrio* and *Photobacterium* outside the Harveyi and Splendidus clades, as well as in the species *Halomonas,* as deduced by their amino acid sequence similarity with sequences extracted from the NCBI database on June 27, 2024 (Table S2).

### Existence of both ancient and mobile *ept*A paralogues in the Harveyi clade

In order to determine the potential risk of *ept*A gene transfer from *Vibrionaceae*, we analyzed the genomic environment of *ept*A genes in 24 243 genomes. The majority of *ept*A genes were single copy (Fig. S5; 19 118/24 243 genomes)*,* located on chromosomes and we did not detect any insertion sequences, phage fragments, or plasmid fragments in the proximity of −4/+4 genes around these single copy *ept*A genes, supporting the ancient acquisition of *ept*A genes in *Vibrio*. However, we also identified 2543 genomes carrying 2 copies and 13 genomes carrying 3 copies of *ept*A (Fig. S5). Some of these additional copies showed sign of recent mobilization as indicated by the presence of transposases/integrases near 137 *ept*A genes from *Vibrionaceae* (0.57% of *ept*A sequences) and 22 *ept*A genes predicted to be carried on a plasmid (Dataset 2).

**Figure 4:**
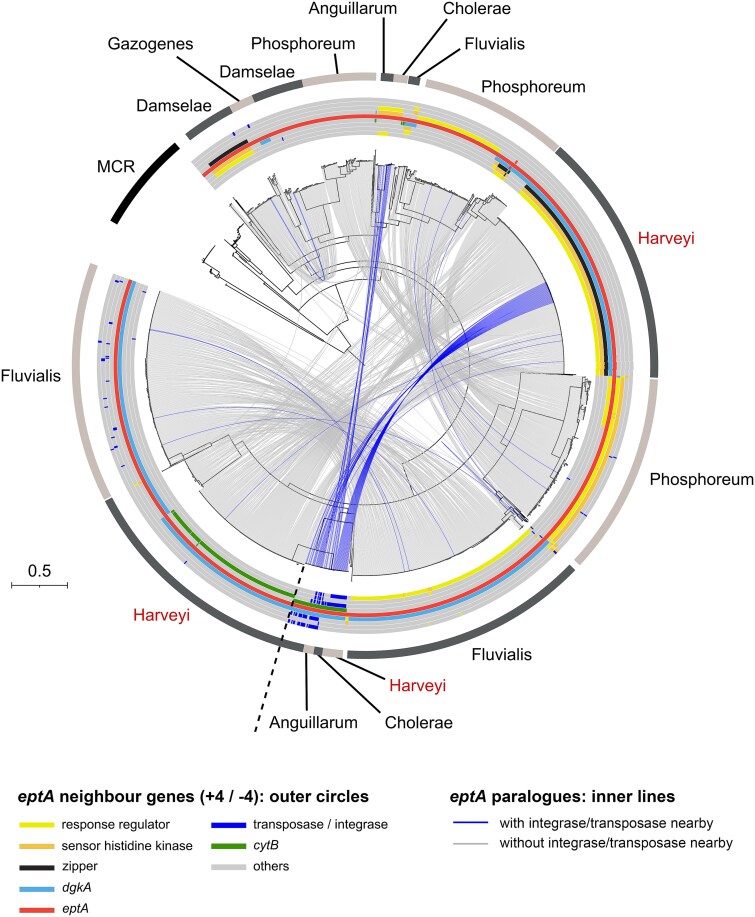
Evidence of gene mobilization in *ept*A paralogues from *Vibrionaceae* phylogeny of *ept*A paralogues for genomes containing multiple *ept*A genes, focusing on the distribution of *ept*A genomic environments (−4/+4 genes). The *ept*A phylogenetic tree was constructed using FastTree with the LG model, and visualization was performed with iTOL. To simplify the figure*, Vibrio vulnificus*, which contains two conserved *ept*A paralogues (see Fig. S5) was not included. All paralogues are connected by inner grey lines, while inner blue lines indicate paralogues associated with integrase/transposase in their −4/+4 environment. The outer circles represent genes in the genomic environment of *ept*A genes Conserved gene families include response regulators, sensor histidine kinases, *dgk*A, glycine zipper family proteins, transposases/integrases, and cytochrome B family proteins. The outermost circle (alternating dark and light grey lines) indicates the *Vibrio* clade according to *Vibrio* clade 3.0 [44]. MCR sequences are shown with a back line. *Vibrio* clades are labeled near *eptA* paralogues, with the Harveyi clade indicated in red.

In species of the Harveyi clade, conserved *ept*A copies (e.g. *ept*A-1, −2 and − 3) as part of the *rst*A-*rst*B-glycine zipper-*dgk*A*-ept*A genomic environment showed no trace of mobile genetic elements in close vicinity (Figs 2−3). Some Harveyi species such as *V. parahaemolyticus* also harbored a second copy of *ept*A in a distinct genomic environment (*cyt*B-*pep*SY-*ept*A-*dgk*A) with no evidence of mobility (Fig. 4). Remarkably, additional *ept*A paralogues with transposases or integrases in close proximity (−4/+4 genes) were found in a *cyt*B*-ept*A*-dgk*A genomic environment (Fig. 4). They showed much closer similarity with *ept*A genes from other *Vibrio* species (*V. cholerae, Vibrio anguillarum*) than with the conserved *ept*A copy from the Harveyi clade (Fig. 4). These results strongly suggest a recent mobilization of *ept*A paralogues between the Harveyi, Cholerae and Anguillarum clades. Similar events of putative *ept*A mobility were also observed within the clade Fluvialis where putative mobile paralogues were found in a PAP2-*dgk*A-*ept*A genomic environment (Fig. 4). Altogether, these results highlight the existence of an ancestral *ept*A gene acquisition in *Vibrionaceae*, which evolved with the *Vibrionaceae* lineage. This conserved gene copy was accompanied by rarer and more recent *ept*A gene mobilizations within and between *Vibrio* clades.

### EptA variants show distinct geographic distribution in Europe

We next examined the geographic distribution of EptA sequences in culturable bacteria isolated from oysters along European coasts. The most common EptA protein variants identified in our sampling clearly clustered according to geographic locations (Fig. 2). Specifically, a major Mediterranean cluster dominated by EptA-1 to -3 (Harveyi clade) included sequences obtained from the EBRO and THAU sites. This drives the significant geographic structuring between the Mediterranean and North Sea sites (PERMANOVA all sites: F_2,68_ = 3.032, P *P* < .001, THAU vs. SYLT: F_1,34_ = 3.108, *P* = .007, EBRO vs. SYLT: F_1,50_ = 4.799, *P* < .001, THAU vs. EBRO: F_1,52_ = 0.900, *P* = .513). Within this Mediterranean cluster EptA-1 and EptA-3 were the more frequently detected variants. Outside this Mediterranean cluster, EptA-4 from the Splendidus clade showed a much weaker geographical structuring and no significant association with a given environment was found (PERMANOVA F_2,16_ = 2.888, *P* = .076; Fig. 2).

### EptA variants from Harveyi but not Splendidus clade are associated with intrinsic colistin resistance

To investigate the potential role of EptA variants from the Harveyi and Splendidus clades in colistin resistance, we focused on bacterial pools positive for *ept*A genes. We conducted whole genome sequencing (WGS) on 9 randomly chosen strains isolated from Thau (included in pools THAU P3–7 and THAU P1–5, Table S1), which displayed resistance to 5 μg/ml colistin in Zobell medium. Among the 9 selected strains, 6 strains were from the Harveyi clade. The *ept*A-1 gene was carried by *Vibrio jasicida* TH21_20A_OE8, *V. owensii* TH21_37_OE7 and *Vibrio* sp. TH21_20A_OB7. The *ept*A-3 gene was carried by *V. alginolyticus* TH21_37A_OE12, *V. alginolyticus* TH21_37_OE9 and *V. alginolyticus* TH21_37A_OE10 (Fig. 4, Table S4). The remaining three strains lacked the *ept*A gene (see the paragraph on other mechanisms of colistin resistance below).

**Figure 5:**
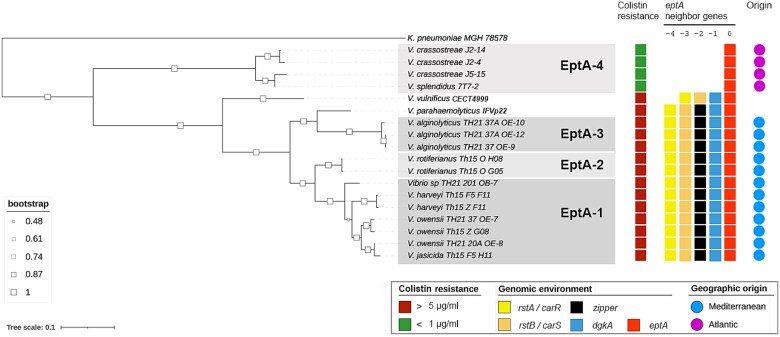
Colistin resistance correlates with EptA polymorphism and *ept*A genomic environment. A phylogenetic tree of EptA amino acid sequences was generated through maximum likelihood analysis of deduced MCR/EptA amino acid sequences using PhyML v-3.0 (https://ngphylogeny.fr/tools/) with the WAG model and 100 bootstrap runs. The tree was constructed using sequences from nine *vibrio* strains isolated in the present study, ten *vibrio* strains isolated over the past ten years in France that harbor an *eptA* gene, and two pathogenic strains of *Vibrio vulnificus* and *Vibrio parahaemolyticus*, also harboring an *eptA* gene. Red and green empty squares indicate strains resistant to 5 μg/ml or susceptible to 1 μg/ml colistin, respectively, as phenotyped by MIC determination in Zobell medium. Colored plain squares indicate conserved genes present at the vicinity of *ept*A: *rst*A/*car*R, *rst*B/*car*S, *dgk*A, and glycine zipper family protein. Numbers indicate the position of the upstream genes relative to EptA.

To enrich our collection of strains with *eptA* gene variants to be screened for colistin resistance we explored 20 genomes of *Vibrio* strains collected over the past ten years in French oyster farms. We identified *ept*A-1 and *ept*A-2 in four and two strains of the Harveyi clade, collected in Thau, respectively. We also identified *ept*A*-*4 from the Splendidus clade in 4 strains collected in Brest (French Brittany; Fig. 5, Table S4). We also included in our collection 2 *Vibrio* strains carrying an *ept*A gene with sequenced genomes and known pathogenic potential. The first was *V. parahaemolyticus* strain IFVp22 [54]: it harbors the *ept*A gene variant from the species in the Harveyi *rst*A-*rst*B-glycine zipper-*dgk*A-*ept*A genomic environment. The second, from outside the Harveyi clade, was the zoonotic *V. vulnificus* CECT4999 [55], which harbors a distinct *ept*A gene variant in a *car*R*-carS*-*dgk*A-*ept*A genomic environment, where *car*RS (also known as *vpr*AB) is homologous to *rst*AB. Strain phenotypes were then determined by screening for resistance to 5 μg/ml or susceptibility to 1 μg/ml colistin in Zobell medium.

Within these 16 strains, resistance to colistin could be predicted by their *ept*A variant makeup (Fig. 5). *Vibrio* strains with *ept*A-1 to −3 were resistant to >5 μg/ml colistin in Zobell medium, similar to the pathogenic strains *V. parahaemolyticus* IFVp22 and *V. vulnificus* CECT4999. In contrast, strains carrying *ept*A*-*4 were susceptible to colistin at a concentration ≤1 μg/ml in the same conditions (Table S4, Fig. 5), suggesting that *ept*A-1 to *−*3, but not *ept*A-4 confer resistance to colistin. Moreover, the *dgk*A gene is consistently found adjacent to *ept*A in resistant strains (Fig. 5) which characterizes Harveyi and Vulnificus clades in general (Fig. 5). In contrast, the *dgk*A gene is absent from the *ept*A genomic environment in the 4 colistin-susceptible strains harboring *ept*A-4 (Fig. 5) and in the Splendidus clade in general (Fig. 3A). In strains of the Harveyi clade, we also noted the conservation of the *rst*A-*rst*B two component signal transduction system (*car*R-*car*S in the Vulnificus clade), located 1084-bp upstream of the *ept*A gene and 706-bp of the *dgk*A gene (Figs 3 and 5). A CDS encoding a potential glycine zipper protein separated *rst*A-*rst*B from the *dgk*A*-ept*A operon in the Harveyi clade (Fig. 3).

### *dgk*A is required for *ept*A-mediated colistin resistance in the Harveyi clade

The co-occurrence of *dgk*A and *ept*A in resistant strains of the Harveyi clade prompted us to test their role in resistance to colistin. Gain of function assays were performed by cloning genes of interest into the pBAD-TOPO TA expression vector used to transform a colistin-susceptible strain of *E. coli.* Cloning was conducted in *E. coli* TOPO10. Basically, we cloned the naturally occurring *dgk*A-*ept*A-1 and *ept*A-4 under the control of a P_BAD_ promotor. In addition, we cloned *ept*A-1 alone and *dgk*A (from *eptA*-1) alone under the control of P_BAD_. Among these four constructs, only *dgkA-eptA-1* increased *E. coli* TOPO10 resistance to colistin (MIC >16 μg/ml in Zobell medium) upon promotor induction. In contrast, the other three constructs did not impact the resistance of *E. coli* TOPO10 to colistin (MIC = 0.25 μg/ml in Zobell medium; Table 2). The result was confirmed in cation-adjusted Muller-Hinton (MHCA) medium (European Committee on Antimicrobial Susceptibility Testing, EUCAST conditions) where only *ept*A*-dgk*A-1 expression increased the MIC of colistin from 0.5 to 4 μg/ml (Table 2). This demonstrates that neither *ept*A*-*1 nor *ept*A*-*4 alone can confer resistance to colistin. Instead, it shows that *dgk*A and *ept*A-1 act together to confer Col-R, in agreement with the conserved genomic environment of *ept*A gene variants 1, 2 and 3 isolated from Mediterranean coastal environments.

**Table 2:** Role of *ept*A variants, *dgk*A and *rst*A in resistance to colistin. MICs were determined by the microdilution assay in the range of 0.125–16 μM colistin.

Strain	Genotype	MIC (μg/ml)	
MHCA			Zobell
*E. coli* TOP10	Wild-type	0.5	0.25
*E. coli* IHPE 10466	TOP10 pBAD-TOPO*-ept*A*-1* ^*^	0.5	0.25
*E. coli* IHPE 10471	TOP10 pBAD-TOPO*-dgk*A ^*^	0.5	0.25
*E. coli* IHPE 10469	TOP10 pBAD-TOPO*-dgk*A*-ept*A*-1* ^*^	**4**	**> 16**
*E. coli* IHPE 10467	TOP10 pBAD-TOPO*-ept*A*-4* ^**^	0.5	0.25
*V. harveyi* IHPE 390 (Th15_F5-F11)	Wild-type *rst*A*-rst*B-glycine zipper*-dgkA-ept*A*-1*	**8**	**> 16**
*V. harveyi* IHPE 10451	Th15_F5-F11 Δ*rst*A	0.5	2
*V. harveyi* IHPE 10453	Th15_F5-F11 Δ*rst*A pMRB*-rst*A	**8**	**> 16**
*V. harveyi* IHPE 10452	Th15_F5-F11 Δ*rst*A pMRB*-gfp*	0.5	2
*E. coli* O6 (ATCC 25922)		1	1

^*^Cloned from *Vibrio owensii* Th15_Z_G08 (IHPE 303).

^**^Cloned from *V. tasmaniensis* 7T7_2 (IHPE 20).

### *rst*A/B controls colistin resistance mediated by *dgk*A*-ept*A in the Harveyi clade

**Figure 6:**
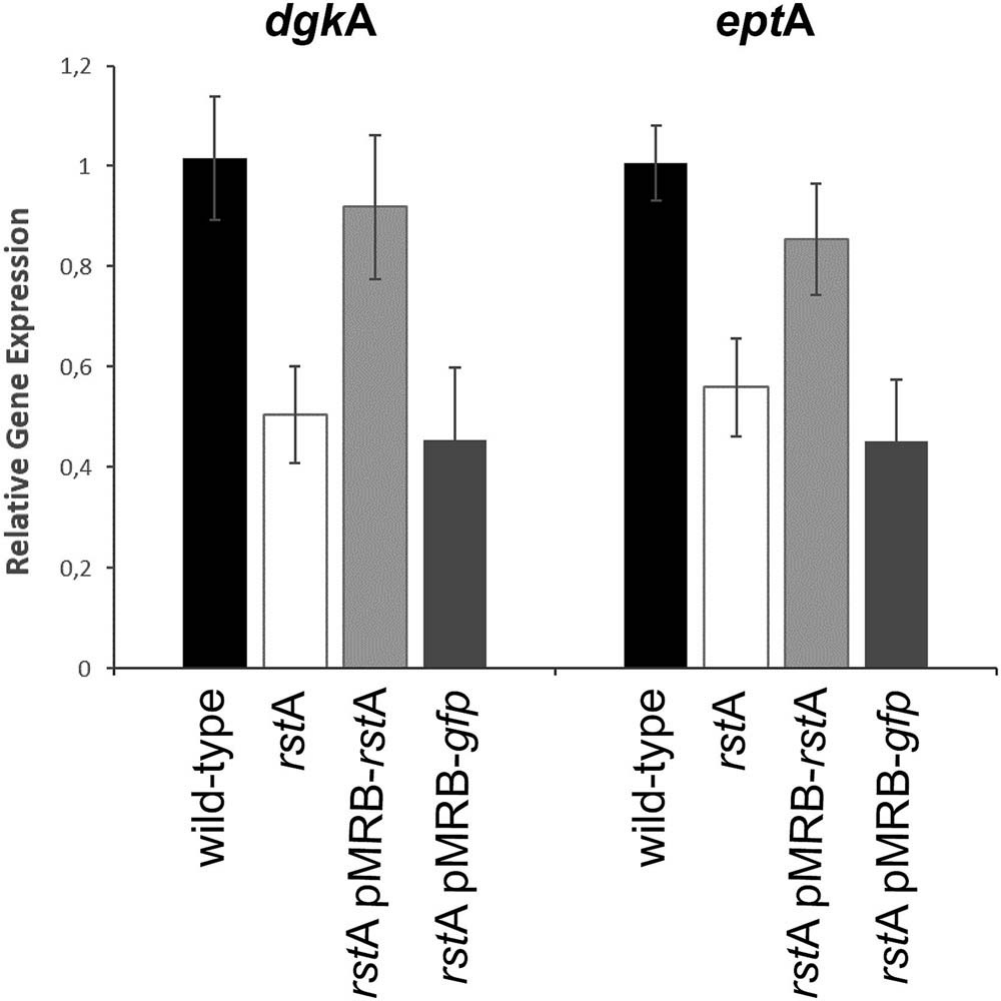
*dgk*A and *ept*A gene expression is controlled by *rst*A. Expression of *dgk*A and *ept*A genes was quantified in wild-type *V. harveyi* Th15_F5-F11 and its *rst*A isogenic mutant. The *rst*A-deletion mutant and the mutant complemented with a pMRB-gfp plasmid showed a significant decrease in expression for *dgk*A (estimate = −0.511 ± 0.173, t = −2.963, *P* = .021 and estimate = −0.561 ± 0.193, t = −2.910, *P* = .023) and *ept*A (estimate = −0.446 ± 0.136, t = −3.271, *P* = .014 and estimate = −0.553 ± 0.153, t = −3.623, *P* = .008), whereas complementation with *rst*A restored wild type expression levels (*dgk*A estimate = −0.0978 ± 0.172, t = −0.567, *P* = .589 and *ept*A estimate = −0.151 ± 0.136, t = −1.109, *P* = .304). Data were normalized using two housekeeping genes.

We finally tested the potential role of the conserved *rst*A/B two-component system in the expression of colistin resistance in Mediterranean *Vibrio* strains resistant to colistin. For that, we performed *rst*A deletion by allelic exchange in *V. harveyi* strain Th15_F5-F11, which harbors the conserved *rst*A-*rst*B-glycine zipper-*dgk*A*-ept*A1 gene cluster and resists to 16 μg/ml colistin in Zobell medium (Table 2)**.** The Col-R phenotype was significantly compromised in the *rst*A deletion mutant, since growth was fully inhibited in the presence of 2 μg/ml colistin (Table 2). Moreover, complementation with the pMRB-*rst*A plasmid was sufficient to restore full growth at 16 μg/ml colistin (Table 2). Similarly, in EUCAST conditions, the resistance phenotype of the wild-type Th15_F5-F11 (MIC = 8 μg/ml in MHCA medium), changed to susceptible in the *rst*A deletion mutant (MIC = 0.25 μg/ml in MHCA medium) and was fully restored in the *rst*A complemented strain, but not using the complementation control plasmid with *gfp* (Table 2). To demonstrate that the loss of the resistance phenotype is due to an altered expression of *dgk*A and *ept*A in the *rst*A deletion mutant, we quantified the transcripts of *dgk*A and *ept*A by RT-qPCR in the wild-type, mutant, and complemented background. As anticipated, the expression of *dgk*A and *ept*A genes exhibited a significant decrease in the *rst*A deletion mutant (Fig. 6). On the other hand, expression levels were not significantly different between the wild-type *V. harveyi* Th15_F5-F11 strain and its isogenic *rst*A mutant complemented with pMRB-*rst*A (Fig. 6). Such a functional complementation was not observed with the pMRB-*gfp* control plasmid. This demonstrates that *rst*A/B controls the Col-R phenotype of *V. harveyi* Th15_F5-F11 through the expression of the *dgk*A*-ept*A1 operon*.*

### Other mechanisms of colistin resistance were found in *Vibrio*

While the *rst*A*-rst*B*-glycine zipper-dgk*A*-ept*A gene cluster was conserved in 6 of the 9 sequenced colistin-resistant isolates from Thau (66,6%), three resistant isolates from our European sampling lacked both the *ept*A and *mcr* genes (*Vibrio sp.* TH21_20_OG1, TH21_20_OH4 and TH21_20A_OC7). In these strains, we detected a well-known colistin resistance mechanism, encoded by *arn*BCADTEF (transfer of L-arabinose onto Lipid A), along with the *pho*P/Q two-component system known to regulate the expression of *arn*ABCDEFT in a broad number of bacterial species [15] (Fig. S6). These three isolates exhibited an average nucleotide identity (ANI) of 91% with *Vibrio variabilis* strain CAIM1454, a marine bacterium previously isolated from the cnidarian *Palythoa caribaeorum* [56]. Next to *pho*P/Q and *arn*ABCDEFT, we further detected a range of other orthologs of known colistin resistance genes, such as *pmr*A/B (= *bas*S/R), *crr*A/B, *sox*R, *tol*C, *kpn*E, *mpr*F, and *ug*D genes in the pool sequencing libraries from Thau, Sylt and Ebro [15]. While we have not functionally characterized these genes, the high prevalence of colistin resistance in the absence of *eptA*-1-2-3 suggest the presence of a substantial diversity of known and probably also novel resistance mechanisms.

## Discussion

This study reveals that *eptA* genes, which encode phosphoethanolamine transferases, are both abundant and diverse in culturable bacteria isolated from European coastal environments and are widely distributed across *Vibrionaceae*. We specifically show that ancient copies of *eptA* have evolved within distinct genomic environments specific to each *Vibrio* clade and contribute to intrinsic colistin resistance in *Vibrionaceae*. In addition, a number of *ept*A paralogues exhibit signatures of recent mobilization within *Vibrio*, highlighting the need for increased surveillance.

While *eptA* genes conferring colistin resistance had previously been described in a number of *Vibrio* species, little was known about their distribution in coastal environments nor within the *Vibrionaceae* family. Here we demonstrated a high diversity of *eptA* genes circulating in European coastal waters (France, Germany, Spain), with the most abundant variants found in *Vibrio* species belonging to the Harveyi and Splendidus clades. Among them, we identified four novel *ept*A gene variants referred to as *ept*A*-*1 to −4 in this study. EptA-1 to −3 were carried by colistin-resistant strains of the Harveyi clade assigned to the species *V. alginolyticus*, *Vibrio campbellii, Vibrio diabolicus, V. harveyi*, *V. jasicida*, *V. owensii*, and *V. rotiferianus.* In contrast EptA-4 was carried by susceptible strains of the Splendidus clade (species *V. splendidus* and *Vibrio crassostreae*). These two *Vibrio* clades naturally colonize oysters, and several species such as *V. harveyi* and *V. crassostreae* cause infections in oysters [57, 58]. A remarkable contrast was observed in the geographic distribution of *ept*A gene variants at a European scale, with the active forms (*eptA*-1 to -3) only being detected in the Mediterranean area (Ebro, Spain and Thau, France), suggesting an adaptive advantage responding to specific selection pressures in that environment. However, since these genes were carried by *Vibrio* of the Harveyi clade, which are adapted to warmer seawater temperatures, the distribution of these *ept*A variants likely reflects the geographic range of *Vibrio* species along European coasts. This does not rule out the possibility that environmental factors also select for *ept*A-mediated Col-R in the Mediterranean coastal environments. No division was observed in the geographic distribution of *ept*A*-*4 (Splendidus clade), which were found in all three European environments along with a number of unknown *mcr* genes. Our data also showed that two weeks were sufficient for oysters to capture a number of Col-R bacteria containing resistance genes specific to each European environment. This finding has implications for the potential transfer of AMR across Europe by oyster transport, a common and still unregulated practice in aquaculture. Such live animal transport has been responsible for the spread at the European scale of oyster pathogens including *Vibrio* [59].

In the Harveyi clade, we described a novel *ept*A genomic environment where *ept*A is co-transcribed with *dgk*A under the control of the RstAB two-component signal transduction system, whose environmental triggers remain unknown. Co-expression of the *dgk*A-*ept*A-1 operon was required for Col-R, in agreement with recent results indicating that *dgk*A is needed for the *ept*A or *mcr*-1-mediated resistance to polymyxins in *E. coli* [60], as well as in environmental isolates carrying *mcr-3* and *mcr-*7 [61]. The underlying mechanism involves the detoxifying effect of DgkA, a diacylglycerol kinase that recycles diacylglycerol, a dead-end metabolite of MCR/EptA proteins, into useful precursor molecules. DgkA plays a crucial role in Col-R by preventing the toxicity of MCR/EptA by-products from inhibiting bacterial growth (for review see [62]). We also showed that co-expression of *dgk*A-*ept*A-1 is controlled by RstAB, which enables bacteria to detect and respond to environmental fluctuations [63–65] and triggers adaptive responses for bacterial survival [64–66]. Until now, in *Vibrio* RstAB had been shown to control motility, adhesion, biofilm formation and hemolytic activity [67]. In *Photobacterium damselae*, RstAB does not control the *eptA*-mediated resistance to colistin [68], but the homologous two-component system (TCS) *carRS* complex, that also controls *eptA* expression and Col-R in *V. vulnificus* and responds to colistin, divalent cations, bile salts, and pH variations [29]. A distinct and still uncharacterized TCS is located at positions +1 and + 2 near the *eptA* gene in *V. cholerae*, where *eptA* expression responds to the acidic conditions encountered in the human gut [27]. Overall, this suggests that *eptA* expression responds to a diverse range of environmental cues relevant to the biology of its *Vibrionaceae* host species. Unlike in the Harveyi clade, the conserved *eptA*-4 copy carried by *Vibrio* of the Splendidus clade, appears to have lost its ability to confer colistin resistance, as also reported for *ept*A from the classical *V. cholerae* strain O395 [27]. Remarkably, like the Splendidus *eptA*-4 copy, the *ept*A copy from *V. cholerae* strain O395 has evolved in a genomic environment lacking *dgk*A [27]. It also lacks a two-component regulatory system. The conservation of the *eptA*-4 gene in the Splendidus clade may indicate that EptA proteins have evolved different specificities/functions along the *Vibrio* phylogeny, although we cannot rule out the possibility that the gene was not activated under our experimental conditions.

Ancestral copies of *eptA* were found widely distributed in *Vibrionaceae* suggesting they contribute significantly to intrinsic colistin resistance in these bacteria. First, from our phylogenetic studies on nearly 30.000 *Vibrionaceae* genomes, we showed that only a very limited number of species (e.g. *Vibrio natriegens)* have lost this gene during evolution. Second, despite sequence identities as low as 43% across the *Vibrio* phylogeny, many *EptA* variants retain a conserved role in conferring colistin resistance, as shown here for EptA-1 from the Harveyi clade in the EUCAST conditions, and reported elsewhere for member species of the *Vibrio* clades Cholerae, Vulnificus, and Fisheri [27, 29, 30]*.* This gives some hints about the functional role and selective pressures encountered by this gene in nature. In the marine environment, various microorganisms, including bacteria produce cationic lipopeptides. For example, species of *Pseudoalteromonas* are members of the oyster microbiota and the lipopeptides they produce (called alterins) are structurally and functionally similar to polymyxins [69]. Moreover, marine animals such as oyster [70] and squid [30] produce cationic antimicrobial (lipo)peptides and proteins as a mechanism of immune defense and to control their microbiota. Like polymyxins and alterins, some of them target the lipopolysaccharide of Gram-negative bacteria [71]. Not surprisingly, Lipid A modifications have evolved as defense mechanisms against cationic antimicrobial peptides, both in pathogens and commensals to circumvent the antimicrobial response of their animal host and competing members of the host microbiome [72]. The importance of *ept*A-mediated colistin resistance in the success of host colonization was clearly demonstrated in the squid symbiont *V. fisheri* [30]. An almost identical gene (>98% sequence identity) was sampled two times within this study in oysters from the Thau and Ebro sites. It is likely that bacterial species that live in close association with marine animals like oysters such as *Vibrio* members of the Harveyi clade [57], have also evolved such resistance mechanisms. We also demonstrated here that conserved copies of *eptA* have evolved within *Vibrio* species in specific genomic environments, further supporting the hypothesis of an ancient acquisition of the *eptA* gene in *Vibrio* phylogenetic history and little to no interspecific horizontal gene transfer since the differentiation of *Vibrio* species. The diversity of genomic environments observed for *eptA* variants is specific to *Vibrio* clades. As we showed here for *V. harveyi*, these genomic contexts are key determinants of EptA expression and activity. The presence in many *ept*A genomic contexts of clade-specific two-component regulatory systems suggests that *eptA* gene expression responds to distinct environmental triggers in different *Vibrio* clades.

Importantly, we also found recent mobility events for certain *eptA* paralogues. Indeed, beyond the ancient *ept*A copy inherited by the majority of *Vibrionaceae* species, we found that a number of species from the clades Harveyi, Cholerae, Anguillarum and Fluvialis harbor mobile *ept*A paralogues. Their genomic environment differs from the clade-specific ancient *ept*A copy, indicating they are likely expressed in response to distinct environmental signals. Although representing less than 1% of the *ept*A genes identified in *Vibrio*, the mobile *ept*A paralogues are surrounded by transposases and integrases and show signs of recent mobility both at an intra-clade and at an inter-clade level. The presence of transposases/integrases directly flanking *cytB-eptA-dgkA* in the clades Harveyi, Cholerae, and Anguillarum is reminiscent of transposases bordering several *mcr* genes, often enclosing them from both sides (e.g., *mcr-3, mcr-5, mcr-8*, and *mcr-9*) [18]. The mobilization of *mcr-1-pap2* as part of the composite transposon Tn*6330* was experimentally demonstrated [73]. Remarkably the co-occurrence of *ept*A with a *dkg*A gene in its direct neighborhood, suggests functionality [62]. This deserves particular attention since such mobile genetic elements significantly increase the risk of environmental capture, as this happened for the *mcr-*1 gene carried by a plasmid and now circulating in human pathogens.

## Conclusion

Our exploration of the genetic basis of Col-R focused on *mcr/ept*A genes, which have a putative origin in aquatic environments and currently raise concerns in a clinical context worldwide [74, 75]. We found a high diversity of *eptA* variants, but only a limited number of *mcr* variants in bacteria isolated from oysters in European coastal waters. An ancient *eptA* gene copy was highly prevalent in *Vibrio* supporting a key role in *Vibrio* intrinsic resistance to colistin including strains from species responsible for most human pathologies (*V. cholerae*, *V. parahaemolyticus*, *V. vulnificus*, *V. alginolyticus*). Most often, these species harbor a *dgkA-eptA* operon under the control of a two-component regulatory system (RstAB in the Harveyi clade or its homolog CarRS in the Vulnificus clade). In European coastal environments, the newly described Harveyi *ept*A genomic environment was identified in Mediterranean strains of *V. alginolyticus* and *V. harveyi,* which thrive in warm seawaters. The chromosomal location of *ept*A, the absence of mobile genetic elements at the vicinity of the *dgk*A*-ept*A genes and the evidence that the *ept*A gene polymorphism has evolved with the *Vibrio* lineage argue in favor of a low risk of horizontal gene transfer from *Vibrio* to other bacterial genera. However, the identification of mobile *ept*A paralogues in *Vibrio* genomes should warn us about a risk of mobilization outside *Vibrionaceae*. Further studies will be needed to characterize the risk associated to other Col-R mechanisms present in oyster-associated bacteria. In the context of global warming, where *Vibrio* are already causing an increasing number of human disease cases in Europe [76], we expect colistin-resistant *Vibrio* species of the Harveyi clade to proliferate. An in depth understanding of the spread of Col-R and of ecological factors interfering with it in coastal areas, and of its persistence and selection in the oyster microbiome is fundamental for the preservation of sustainable system of oyster aquaculture in oceans exposed to global warming [23].

## Supplementary Material

Saad_J_et_al_ISME_Comm_revised_III_Datasets_1_and_2_ycaf055

Saad_J_et_al_ISME_comm_revised_III_Supp_Data_ycaf055

## Data Availability

Targeted gene sequences (*eptA*) and pool-sequencing raw data were deposited at GenBank under accession numbers OR578979 to OR579029 and SAMN37810832 to SAMN37810840, respectively. Genome raw data and assemblies were deposited at the European Nucleotide Archive (ENA) under project accession no. PRJEB67316 (ERR12116510 to ERR12116518) and are available on MicroScope plateforme MaGe (“Magnifying Genomes”) https://mage.genoscope.cns.fr/.

## Code availability

Software’s and databases used in this paper are openly available in Galaxy Open platform hosted by our institute https://galaxy-datarmor.ifremer.fr/. Specific source codes for antimicrobial resistance gene identification are also available on GitHub https://github.com/lakinsm/meta-marc.
