# Supplementary material for: Vibrio are a potential source of novel colistin-resistance genes in European coastal environments
Source: ISME Commun. 2025 May 5;5(1):ycaf055. doi: 10.1093/ismeco/ycaf055 (PMC12064563; doi:10.1093/ismeco/ycaf055)
Supplement: Saad_J_et_al_ISME_comm_revised_III_Supp_Data_ycaf055 [file saad_j_et_al_isme_comm_revised_iii_supp_data_ycaf055.pdf]

Supplementary information

Vibrio are a potential source of novel colistin-resistance genes in European coastal environments

Jamal Saad[1], Viviane Boulo[1], David Go.udenège[2], Coralie Broquard[3], Karl B. Andree[4], Manon Auguste[5], Bruno Petton[6,7], Yannick Labreuche[1], Pablo Tris[1], Dolors Furones[4], Augusti Gil[4], Luigi Vezzulli[5], Gianluca Corno[8], Andrea Di Cesare[8], Hugo Koechlin[6], Emilie Labadie-Lafforgue[1], Gaelle Courtay[1], Océane Romatif[1], Juliette Pouzadoux[1], Jean-Michel Escoubas[1], Dominique Munaron[9], Guillaume M. Charrière[1], Eve Toulza[1], Marie-Agnès Travers[1], Caroline Montagnani[1], Mathias K. Wegner[2], Delphine Destoumieux-Garzón[1]*

1. IHPE, Univ Montpellier, CNRS, Ifremer, UPVD, Montpellier, France

2. Ifremer, SeBiMER - Marine Bioinformatics Platform, Brest, France

3. AWI Alfred Wegener Institute – Helmholtz Centre for Polar and Marine Research, Waddensea Station Sylt, List, Germany

4. Institute of Agrifood Research and Technology IRTA, Ebro, Spain

5. Department of Earth, Environmental, and Life Sciences, University of Genoa, 16132 Genoa, Italy

6. LEMAR, Univ Brest, Ifremer, CNRS, IRD, 29280 Plouzané, France

7. Ifremer, Unité Physiologie Fonctionnelle des Organismes Marins, ZI de la Pointe du Diable, Plouzané, France

8. MEG Molecular Ecology Group - Water Research Institute, CNR - National Research Council of Italy, Verbania, Italy

9. MARBEC, Univ Montpellier, CNRS, Ifremer, IRD, Montpellier, France

Table of content

Fig. S1. Bioinformatics analyses for identifying *ept*A and *mcr* gene sequences in Poolseq data. p 3

Fig. S2 The pourcentage of Col-R bacterial isolates across European oyster farms. p 4

Fig. S3. Phylogeny of EptA/MCR sequences based on medium culture and genus identification. p 5

Fig. S4. Multiple sequence alignment of EptA-1 to -4 with EptA orthologs in *Vibrionaceae*. p 7

Fig. S5. Percentage of EptA paralogues in species of *Vibrionaceae*. p 8

Fig. S6. Alternative colistin resistance mechanisms identified in *Vibrio* strains TH21_20_OC7, TH21_20_OH4, and TH21_20A_OG1. p 9

Table S1. Metadata on *mcr/eptA* unique sequences found in Pool sequencing data p 10

Table S2. List of *mcr*/*ept*A unique sequences found in pool sequencing data p 11

Table S3. Amino acid sequence identity between EptA variants. p 13

Table S4. Strains and Plasmids p 14

Table S5. List of primers used in this study p 17

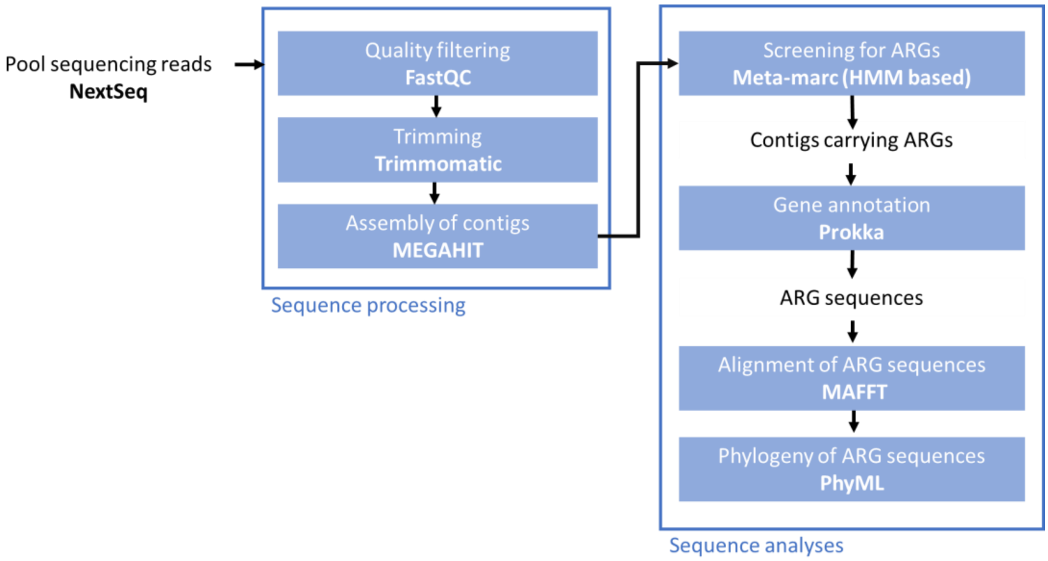

Fig. S1. Bioinformatics analyses for identifying *ept*A and *mcr* gene sequences in Poolseq data.

A

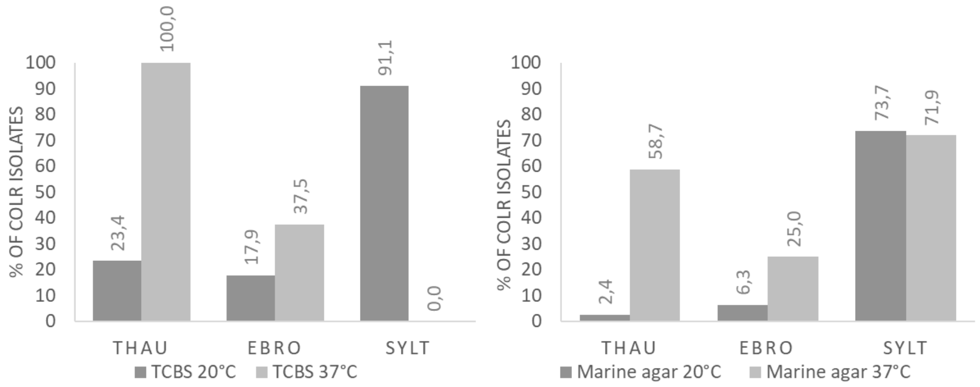

B

	Estimate	Std. Error	z value	Pr(>|z|)
**Intercept**	-4.0118	1.0429	-3.847	0.00012
**Temp37**	**4.3876**	**1.0661**	**4.115**	**3.86e-05**
**MediumTCBS**	**2.8581**	**1.0727**	**2.664**	**0.00771**
**SiteEBRO**	1.3510	1.0855	1.245	0.21328
**SiteSYLT**	**5.0414**	**1.1658**	**4.324**	**1.53e-05**
**Temp37:MediumTCBS**	-0.3747	0.8405	-0.446	0.65571
**Temp37:SiteEBRO**	**-2.8585**	**1.1307**	**-2.528**	**0.01147**
**Temp37:SiteSYLT**	**-4.4789**	**1.2501**	**-3.583**	**0.00034**
**MediumTCBS:SiteEBRO**	-1.7907	1.1590	-1.545	0.12233
**MediumTCBS:SiteSYLT**	-1.5604	1.3025	-1.198	0.23090

Fig. S2 The percentage of Col-R bacterial isolates across European oyster farms.

(A) % Col-R isolates on TCBS (left panel) or marine agar (right panel). (B) Results of a binomial general linear model showing the effect of environmental origin (Site), temperature (temp) and culture medium (Medium) plus their two-way interactions on the proportion of Col-R bacteria.

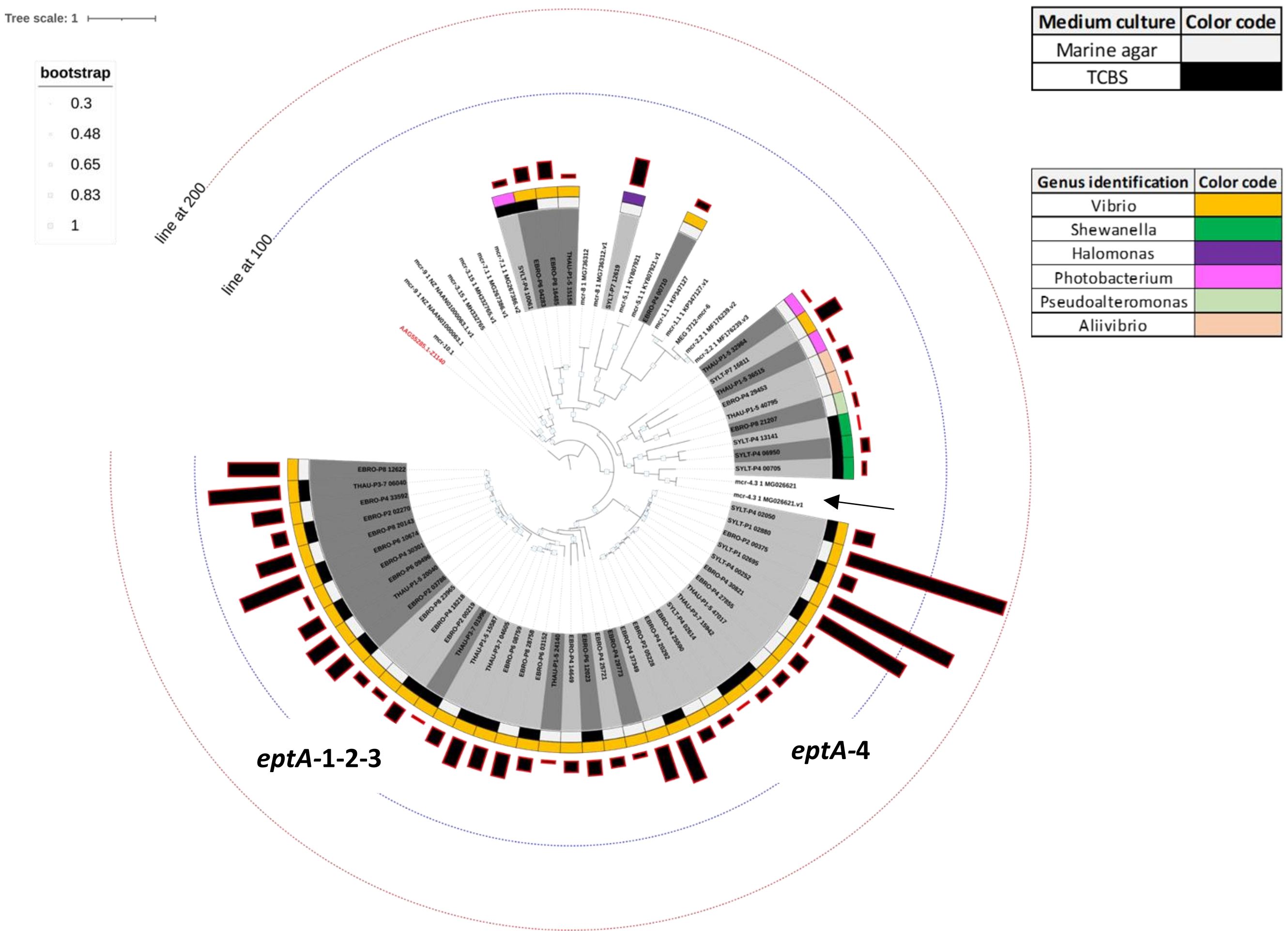

Fig. S3. Phylogeny of EptA/MCR sequences based on medium culture and genus identification.

This figure represents conditions of isolation (back and grey boxes), genus identification (color boxes) and read counts (black bars with a red outline). The phylogenetic tree was generated through maximum likelihood analysis of deduced EptA/MCR amino acid sequences using PhyML V-3.0 with the WAG model and 100 bootstrap runs. Amino acid sequences were extracted from the annotated file generated from pooled sequencing of bacteria isolated from both Marine agar and TCBS medium across three European regions. This figure represents conditions of isolation, genus identification and read counts. Tree was visualized using the iTOL online website. Blue line at 100 and red line at 200 represent the numerical scale of normalization. MCR-4 sequences are indicated by an arrow.

```
EptA-1                    -------MKTIELPTKGLSYVTFTLLLALYFALVVNIPIYKELVHILTNLDQVKIGFIIT   53
EptA-2                    -------MKNITFPYKGISYVTFTLFLALYFALVVNIPIYKELSHILSNLDEVKIGFIIT   53
EptA-3                    -------MKTHNLQQKGISYVTFTFLLALYFALVVNIPIYKELNGIFSSLDNVKIGFIIT   53
EptA-4                    ----------MNLPSLNMSINKLPFVLAVYYLLVINIPLSLELFSIVQASKSESVAFLIS   50
A. fischeri               MLVLSTLISKIKNHKISMNMVSLITIVSLYFAVAFNYPINEKIYQLSQ----GQEIFLFL   56
V. cholerae El Tor        ------MVIMPTFNIKPFSYLQLVFLLAAYFALPLNLPVYAQLAHIFNQSTSLDWGFALS   54
V. vulnificus             ------MNRIKTLFNRECSYTTFTLVLAIYFATIVNLPIYKELFSIISHLDSVKVGFVIS   54
V. parahaemolyticus       -------MKTVDSPKKGIPYVAFTVLLALYFALVVNIPIYKELIGIFSKLDEVKIGFIIT   53
                                               :  .:: *:   .* *:  ::  :       .  * :

EptA-1                    IPIFFFAALNFLFNLFSWPWISKPFFILLLITSGIVSYASYNYGTLFDTDMITNIVETDT   113
EptA-2                    IPIFFFAALNFLFNLFSWPWLSKPFFIVLLVVSSMVSYASYNYGTLFDTDMITNIVETDT   113
EptA-3                    IPIFFLAALNFLFNLFSWPWVGKPFFIILLIISSMVSYASYNYGTLFDSGMIANIAETDS   113
EptA-4                    IPIFFLAAFNFIFQVFNWPIFSKPFFIFLLITSTLVSYSMFNYGIYVDYGMIENVFETNN   110
A. fischeri               TPALLTCAFIIIFSFIAFPYVFKGIVVVLTITSAMAFYAALQYNTMFDYAMIENIFETNV   116
V. cholerae El Tor        IPLFFFLFALNFIFQIFSWPYLFKPFFAVLLVLSALISFAGFQYGVIVDQDMLVNVLETDR   114
V. vulnificus             VPIFFLAALNFLFNLFSWPWLSKPFFAVLLLLSAMVSYAGYNYGTLFDYGMIANIMETDS   114
V. parahaemolyticus       IPIFFFAALNFLFNLFSWPWVGKPFFILLLVVSSLVSYAGYNYGTLFDSGMIANIVETDS   113
                           * ::   *: ::*..: :*  .  * :.  .* : * :   ::   :*.   .*   *: *: **:

EptA-1                    SEASSYFSAYSLIWTVLMGVLPALLVFKVKFQSLKGNWLRFTLTKIVSMLASLVIIAVIA   173
EptA-2                    SEASSYLSAYSVIWTLLMGMIPAALVFKVKLQPLNGQWLRFVLTKVVSMLASLAVIAVIA   173
EptA-3                    SEASSYLSTYSVIWTLLMGGVPAMIVYKVKLQSQRHQLLYFSLTKLVSMLSSLAVIAVIA   173
EptA-4                    GEAASYVSTHSLLWLLVMGGIPSLILLLTKLK--RESWKDFFVWKSIGLLSSLIVIAIIA   168
A. fischeri               DEASSYLSSASIGYLIVFGLIPSILLLKVNIVR-HASWLKELFHRAILMSVAIVGLLLIS   175
V. cholerae El Tor        GEAGSYLTIYSVLWLLGFGLVPALALLFTPIRPE-KSALRFLMKKGLSMLVSVVVIGVIA   173
V. vulnificus             SEAGSYLSAYSVIWTLLMGVVPALLVCKLNLS-LSGSTLHMVAKKAASMLASLAVIAVIA   173
V. parahaemolyticus       SEASSYLSTYSVVWATLMGVIPALIVFKVKLQPQRGQWLRFVLTKLVAMLASLAVIAVIA   173
                          .**.**.:    *: :    :* :*:   :     :     .       :   :  ::  : :*:

EptA-1                    GLYYQDYASVGRNNSYLKKIIIPTQYVYAISSYVKETYLTTPQPYREIGTDAKQSQLALE   233
EptA-2                    GLYYQDYASVGRNNSYLKKVIIPTQYVYSISSYVKAKYLTTPEPYREIGLDAKQSSTALQ   233
EptA-3                    SVYYQDYASVGRNNSYLKKIIIPTQFVYSATGYVKEKYFTTPEPYREIGIDAKQSDSAVK   233
EptA-4                    GLFYKDYVSIGRNNSHIKKMIIPTEYVASTVKYINNTYVKEPIPYQELGLDAQLKPEAK-   227
A. fischeri               VFYFKDYASIGRNNSYLNKMINPAD-AFNTVKYIKNEYLTAPLEYITIGEDAVVTPA---   231
V. cholerae El Tor        GLYYQNYSSVGRNNSSLKKMIIPTHFLYSSFGLVKQRYFTEPMVYQEIGQDAQQKPSAVR   233
V. vulnificus             GLYYQDYASIGRNNTHLKKMIIPTQYVYSATKYVRDTYFSTPQPYRQLGLDAKQSELALQ   233
V. parahaemolyticus       GLYYQDYASVGRNNSYLKKMIIPTQYVYSATSYVKENYLTTPQPYREIGTDAQQSSTALQ   233
                           .::::* *:****: ::*:* *:.         :.   *..  *   *   :* **   .

EptA-1                    QAKEKPTLVVFVLGETARTQNYQINGYDRETNPYTSKLDVISFQDVSSCGTATAVSVPCM   293
EptA-2                    QANGKPTLLVFVVGETARTQNYQLNGYERETNPYTSKLDVVSFKDVASCGTATAVSVPCM   293
EptA-3                    QAQNKPTLLVFVVGETARTQNYQLNGYKRETNPYTSQLDVISFQEVASCGTATAVSVPCM   293
EptA-4                    -QAEKPTLLVFVLGETARVYNYQYYGYEKETNAHTKPYNPIFFSDVQSCGTATAVSVPCM   286
A. fischeri               -KNGKPTLMVILVGETARAQNSAYNGYERETNPYTKDLGLITFQNVTSCGTATAHSLPCM   290
V. cholerae El Tor        QATQKPTLVFFVLGETARVQNYQYLGYPRDTNAYTAPFQPIFFKDVASCGTATAVSVPCM   293
V. vulnificus             QADQKPTLLVFVVGETARSQNYQANGYARPTNQYTNELGMISFQDVSSCGTATAVSVPCM   293
V. parahaemolyticus       QAQDKPTLLVFVVGETARTQNYQLNGYERETNPYTSQLDVISFKDVASCGTATAVSVPCM   293
                              ****:.:::*****  *    ** : ** :*      : *.:* ******* *:***

EptA-1                    FSQLPRTDFDRSTADNQDNALDIMQRAGIDLMWKDNDGGDKGVAHKINKMDVDRSQKNEQ   353
EptA-2                    FSQLTRSDFDRSRADNQDNALDIMQRAGVDLLWKENDGGDKGVAHNITKIEVDRSQKNEL   353
EptA-3                    FSQLTRSEFERTKADNQDNALDIMQRAGVDLIWKENDGGDKEVAHKIKKIEVNRKQTSPL   353
EptA-4                    FSNMNRSNYDRDKAYNQDNVVDIMNRAGIHSIWREHDGGDKAVAHRIKEMTLVAKDSDPL   346
A. fischeri               FSNMTRTSYNKDRANNQDNALDVLTHAGVKAVWIDNDGGDKAVAKNIEKEMITDHSESNL   350
V. cholerae El Tor        FSNMNRQNFDRSRADNQDNVLDILQRAGISLLWKENDGGDKNVAKNIPLKELARDNREGI   353
V. vulnificus             FSNMTRSNFDRPTADNQDNALDILNRAGVSLLWKENDGGDKDVAKNIPLFTVDRSRKDAL   353
V. parahaemolyticus       FSQLTRNQFDRKQADNQDNALDIMQRAGIDLLWKENDGGDKEVAHKIKKIEVDRKQQNAL   353
                          **:: *  .:::  * ****.:*:: :**:  :* ::***** **:.*      :      .

EptA-1                    CNGSTCYDIALLNNLDDQIASMKGNRLVALHLIGSHGPTYFQRYPKDKAFFQPDCPRADI   413
EptA-2                    CNGSTCYDMALLNNLDTQISSMKGNRVIAMHLIGSHGPTYFQRYPKDKAFFQPDCPRADI   413
EptA-3                    CNGQTCYDMALLDHFDQEVADMHGNRVVAMHLIGSHGPTYFQRYPRDKAFFKPDCPRADI   413
EptA-4                    CNNNVCYDTAMLENFEQDTQDLKQDSIIFYHISGSHGPTYFERYPDDHKKFTPDCARADI   406
A. fischeri               CNGSSCYDEILLKGLDKRIAETQGNQVYALHMIGSHGPTYWKRYPKEMAVFGPACNRSDI   410
V. cholerae El Tor        CDGDTCYDIAMLENLDQEIATQQGNRMIFMHFIGSHGPTYFKRYPKEMAVYQPDCPRADI   413
V. vulnificus             CNGSTCYDMALLENFEQDVENLKGNRVLALHLIGSHGPTYFQRYPKDKAAFMPDCPRADI   413
V. parahaemolyticus       CNGQTCYDMALLSDFDQEVSNMNGNRVVAMHLIGSHGPTYFQRYPKEKAFFQPDCPRADI   413
                          *:.. ***  :*. ::      : : :   *: *******::*** :    : * * *:**
```

In the alignment image, the residue T at position 285 in each sequence (position 278 in EptA-4, 282 in A. fischeri; the T of "GTATA") is highlighted in red.

```
EptA-1               ENCSVEQIVNTYDNTIRYTDFVLDQTIAKLKALEDKYNTAMIYVSDHGESLGENGLFLHG   473
EptA-2               ENCSVEQIVNTYDNTIRYTDFVLDQTIAKLKTLEDKYNTAMIYVSDHGESLGENGLFLHG   473
EptA-3               ENCSVEQIVNTYDNTIRYTDLVLAETINKLKALESQYNTALIYVSDHGESLGENGLFLHG   473
EptA-4               ENCTKEEVVNTYDNTILYTDFFLSQAIQKLEKLTDKYNVALMYISDHGESLGENGVYLHG   466
A. fischeri          ENCSDAEITNVYDNTLVYTDYVIAQTVKKLQSYSDKYNVVMLYISDHGESLGENGLYLHG   470
V. cholerae El Tor   ENCSVEQIVNTYDNTIRYSDYVMSQLLAKLDSLQDRYNTALIYISDHGESLGENGLFLHG   473
V. vulnificus        ENCSVEQIVNTYDNTILYTDYVLSQTINKLKALEERYNTALIYVSDHGESLGENGLFLHG   473
V. parahaemolyticus  ENCSVEEIVNTYDNTIRYTDFVLEQTINKLKTLEDKYNTALIYVSDHGESLGESGMFLHG   473
                     ***:  ::.*.****: *:* .: : : **.   .:**..::*:*********.*::***

EptA-1               MPYGLAPDFQKRVPMILWMSPGFKQAKHINTDCLEQEAQQTATHSHDNVFHSLLGIMDVE   533
EptA-2               MPYGLAPDFQKRVPMMLWMSPGFKQAKHINSDCLQQEAQQTATHSHDNVFHSLLGIMDIE   533
EptA-3               MPYGLAPDFQKRVPLLMWLSSGFKQVKQINTDCLSNEAQKAGQYSHDNVFHSLLGVMDVQ   533
EptA-4               MPYSLAPKEQTHVPMILWMSDGFAAQKRISETCLRKAG-KEQSFSHDNLFDSLLGLMDVQ   525
A. fischeri          APYMMAPKEQTHVPWYIWMSDDYANQKGIDKAAVIDNS-ATGDYSHDNLFHTILGLYGVT   529
V. cholerae El Tor   MPYSLAPEYQTRVPLLIWMSSGFSQSKGIDVECLRSNS--ELPYAHQNLFHSLLGVMDVS   531
V. vulnificus        MPYSLAPEYQTKVPLMLWMSPGFAQAKQINADCLKQQAKQTDTYSHDYIFHSLLGVMNVS   533
V. parahaemolyticus  MPYGLAPDFQKRVPLIMWMSPSFKQAKHINTDCLSKEAQNAGKYSHDNVFHSLLGIMDVK   533
                      ** :**. *.:**  :*:* .:   * *.  .: . .     .:*: :*.::**: .:

EptA-1               TDAYDGDLDIFKTCRTS--       550
EptA-2               TNAYDGKLDIFKTCRNS--       550
EptA-3               TQAYDGKLDIFKDCRTTS-       551
EptA-4               TNEYRASQDIFAPCR----       540
A. fischeri          TKAKDNALDIMAN------       542
V. cholerae El Tor   TKAYQANLDLFAKCRTSQS       550
V. vulnificus        TSEYDATLDLFKQCRAQ--       550
V. parahaemolyticus  TQAYDGQLDIFKTCRTVS-       551
                     *.      *::
```

Fig. S4. Multiple sequence alignment of EptA-1 to -4 with EptA orthologs in *Vibrionaceae*.

The alignment was performed using Clustal Omega (1.2.4). The conserved catalytic threonine residue (T) is in shown in red. EptA-1 to -4 sequences were chosen as one representative of each clade (amino acid sequences deduced from poolseq data). The amino acid sequences from previously characterized EptA orthologs were obtained in NCBI database and the accession numbers are: AAW87280.1 (*A. fischeri* ES114), AAF96994.1 (*V. cholerae* O1 biovar El Tor str. N16961), VVMO6_RS15980 (*V. vulnificus* MO6-24/O), and BAC62623.1 (*V. parahaemolyticus* RIMD 2210633).

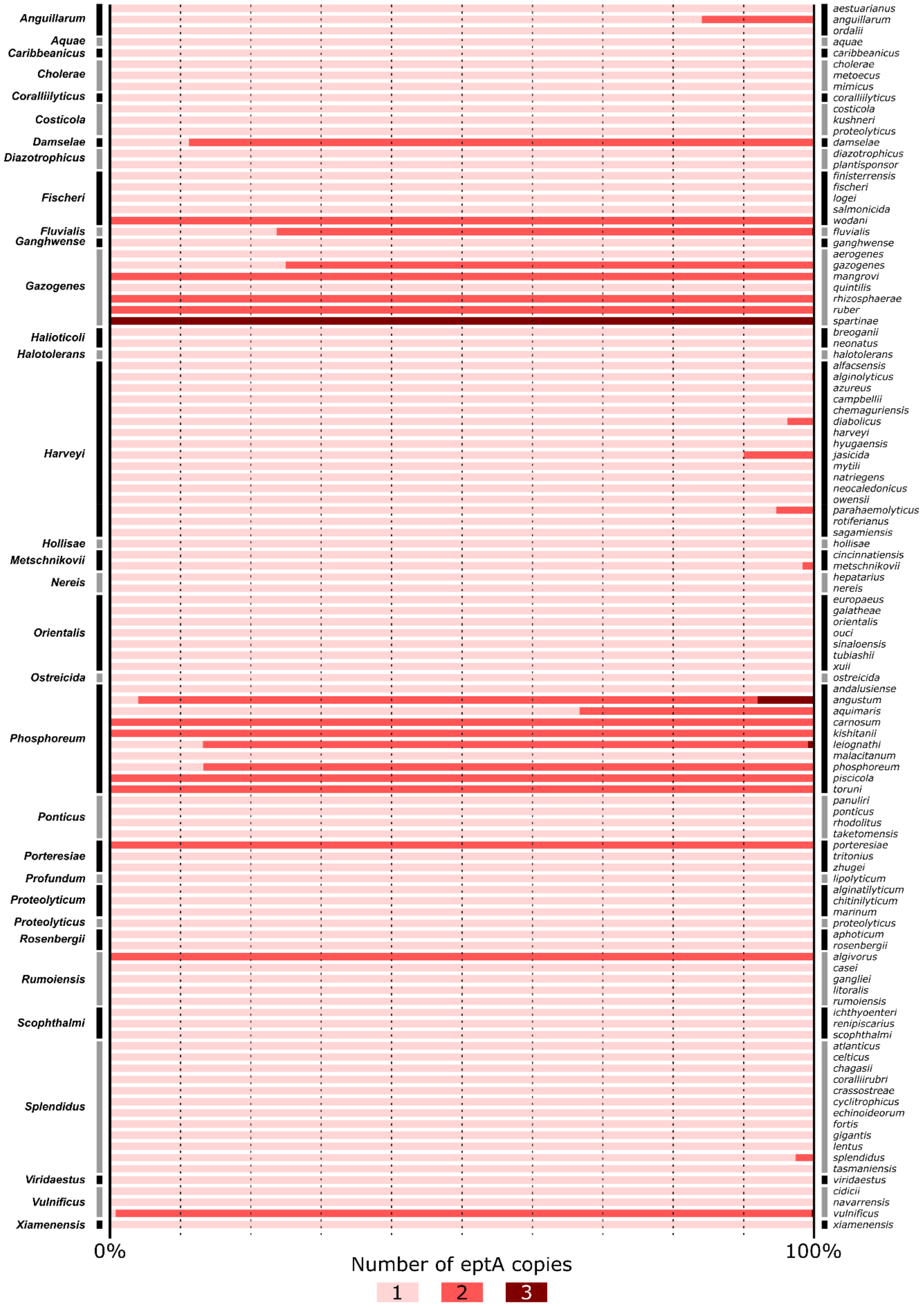

Fig. S5. Percentage of *ept*A paralogues in species of *Vibrionaceae*. Species of *Vibrionaceae* (right) are sorted by clade (left). The color code indicates the number of *ept*A copy per genome: pink (1 gene copy), dark pink (two gene copies), brown (3 gene copies). The scale bar indicates the percentage of genomes carrying 1, 2 or 3 *ept*A copies in each species.

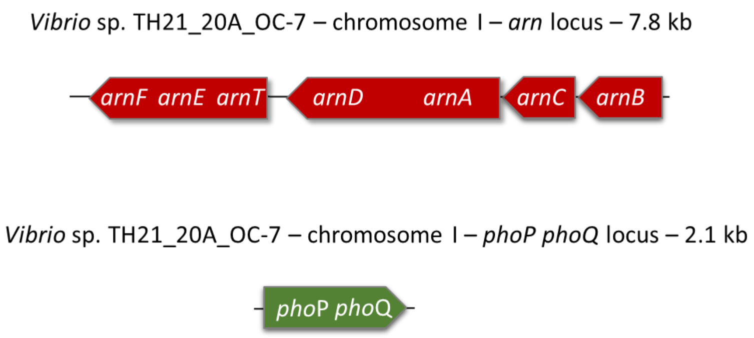

Fig. S6. Alternative colistin resistance mechanisms identified in *Vibrio* strains TH21_20A_OC7, TH21_20_OH4, and TH21_20A_OG1.

Each of these isolates contained both *arn*BCADTEF and phoP/Q genes within their genomes, although they were situated in distinct genomic regions.

Table S1. Metadata on *mcr/eptA* unique sequences found in Pool sequencing data

Sampling sites	GPS coordinates	Sampling date	Culture medium	Temp	No. CFU	Bacterial Pool ID	Total sequenced reads	Accession number	No. unique *mcr/eptA* sequences total (*mcr*; *eptA*)
**THAU**	N: 43º26.058' E: 003º39.878'	18/10/2021	Marine agar	37°C	48	THAU-P1_5	7,53E+07	SAMN37810832	7 (1;6)
				20°C	44				
			TCBS	37°C	6	THAU-P3_7	8,38E+07	SAMN37810833	4 (0;4)
				20°C	48				
**EBRO**	N: 40º37.106880´ E: 0º37.318320´	25/10/2021	Marine agar	37°C	48	EBRO-P8	3,59E+07	SAMN37810834	6 (2;4)
				20°C	47	EBRO-P4	6,88E+07	SAMN37810835	13 (1;12)
			TCBS	37°C	18	EBRO-P6	1,28E+07	SAMN37810836	6 (1;5)
				20°C	36	EBRO-P2	1,44E+07	SAMN37810837	5 (0;5)
**SYLT**	N: 55º 1' 42.539" E: 8º 26' 1.953''	10/01/2022	Marine Agar	37°C	32	SYLT-P7	3,05E+07	SAMN37810838	2 (0;2)
				20°C	19	SYLT-P1	6,57E+06	SAMN37810839	2 (0;2)
			TCBS	20°C	48	SYLT-P4	7,73E+07	SAMN37810840	6 (3;3)
				37°C	0	na	na	na	**0** (0;0)
**Total**					**394**				**51** (8;43)

Table S2. List of the 51 *mcr/ept*A unique sequences found in pool sequencing data

Gene name	*mcr/eptA* sequence ID	Genbank ID	From poolseq library	Sampling site	Genetic environment	Genus based on BlastP best hit	Culture medium	Temp	No. of mapped Reads	Normalised read counts * 10^6
eptA-1	EBRO-P8_12622	OR578988	EBRO-P8	EBRO	*rstA-rstB-Zipper-dgkA-eptA*	*Vibrio*	MA	37°C	2136	60,39
eptA-1	THAU-P3-7_06040	OR578979	THAU-P3-7	THAU	*rstA-rstB-Zipper-dgkA-eptA*	*Vibrio*	TCBS	20+37°C	7010	84,39
eptA-1	EBRO-P2_02270	OR578984	EBRO-P2	EBRO	*rstA-rstB-Zipper-dgkA-eptA*	*Vibrio*	TCBS	20°C	247	17,4
eptA-1	EBRO-P4_33592	OR578987	EBRO-P4	EBRO	*rstA-rstB-Zipper-dgkA-eptA*	*Vibrio*	MA	20°C	2440	35,81
eptA-1	EBRO-P2_03786	OR578981	EBRO-P2	EBRO	*rstA-rstB-Zipper-dgkA-eptA*	*Vibrio*	TCBS	20°C	418	29,44
eptA-1	EBRO-P4_30301	OR578985	EBRO-P4	EBRO	*rstA-rstB-Zipper-dgkA-eptA*	*Vibrio*	MA	20°C	375	5,5
eptA-1	EBRO-P6_09496	OR578982	EBRO-P6	EBRO	*rstA-rstB-Zipper-dgkA-eptA*	*Vibrio*	TCBS	37°C	356	28,11
eptA-1	THAU-P1-5_20040	OR578980	THAU-P1-5	THAU	*rstA-rstB-Zipper-dgkA-eptA*	*Vibrio*	MA	20+37°C	2669	35,89
eptA-1	EBRO-P8_20143	OR578986	EBRO-P8	EBRO	*rstA-rstB-Zipper-dgkA-eptA*	*Vibrio*	MA	37°C	1546	43,71
eptA-1	EBRO-P6_10674	OR578983	EBRO-P6	EBRO	*rstA-rstB-Zipper-dgkA-eptA*	*Vibrio*	TCBS	37°C	958	75,63
eptA-2	EBRO-P2_00219	OR578989	EBRO-P2	EBRO	*rstA-rstB-Zipper-dgkA-eptA*	*Vibrio*	TCBS	20°C	274	19,3
eptA-2	EBRO-P4_18218	OR578990	EBRO-P4	EBRO	*rstA-rstB-Zipper-dgkA-eptA*	*Vibrio*	MA	20°C	492	7,22
eptA-2	EBRO-P8_23965	OR578991	EBRO-P8	EBRO	*rstA-rstB-Zipper-dgkA-eptA*	*Vibrio*	MA	37°C	359	10,15
eptA-3	EBRO-P8_28758	OR578996	EBRO-P8	EBRO	*rstA-rstB-Zipper-dgkA-eptA*	*Vibrio*	MA	37°C	1005	28,41
eptA-3	EBRO-P6_08759	OR578995	EBRO-P6	EBRO	*rstA-rstB-Zipper-dgkA-eptA*	*Vibrio*	TCBS	37°C	333	26,29
eptA-3	EBRO-P6_03152	OR578994	EBRO-P6	EBRO	*rstA-rstB-Zipper-dgkA-eptA*	*Vibrio*	TCBS	37°C	195	15,4
eptA-3	THAU-P1-5_15587	OR578993	THAU-P1-5	THAU	*rstA-rstB-Zipper-dgkA-eptA*	*Vibrio*	MA	20+37°C	1356	18,23
eptA-3	THAU-P3-7_04605	OR578992	THAU-P3-7	THAU	*rstA-rstB-Zipper-dgkA-eptA*	*Vibrio*	TCBS	20+37°C	2751	33,12
eptA-4	EBRO-P2_00375	OR579005	EBRO-P2	EBRO	*eptA*	*Vibrio*	TCBS	20°C	256	18,03
eptA-4	SYLT-P4_02614	OR578999	SYLT-P4	SYLT	*eptA*	*Vibrio*	TCBS	20°C	156	2,03
eptA-4	EBRO-P4_30821	OR579010	EBRO-P4	EBRO	*eptA*	*Vibrio*	MA	20°C	288	4,23
eptA-4	SYLT-P1_02695	OR579002	SYLT-P1	SYLT	*eptA*	*Vibrio*	MA	20°C	1017	159,08
eptA-4	SYLT-P4_00252	OR579000	SYLT-P4	SYLT	*eptA*	*Vibrio*	TCBS	20°C	8823	115,02
eptA-4	EBRO-P2_05228	OR579004	EBRO-P2	EBRO	*eptA*	*Vibrio*	TCBS	20°C	753	53,04
eptA-4	EBRO-P4_25590	OR579006	EBRO-P4	EBRO	*eptA*	*Vibrio*	MA	20°C	612	8,98
eptA-4	EBRO-P4_20292	OR579007	EBRO-P4	EBRO	*eptA*	*Vibrio*	MA	20°C	946	13,89

eptA-4	EBRO-P4_37349	OR579008	EBRO-P4	EBRO	*eptA*	*Vibrio*	MA	20°C	2770	40,66
eptA-4	SYLT-P4_02050	OR579001	SYLT-P4	SYLT	*eptA*	*Vibrio*	TCBS	20°C	1745	22,75
eptA-4	THAU-P3-7_15942	OR578997	THAU-P3-7	THAU	*eptA*	*Vibrio*	TCBS	20+37°C	891	10,73
eptA-4	EBRO-P4_27855	OR579009	EBRO-P4	EBRO	*eptA*	*Vibrio*	MA	20°C	1117	16,4
eptA-4	SYLT-P1_02880	OR579003	SYLT-P1	SYLT	*eptA*	*Vibrio*	MA	20°C	1244	194,59
eptA-4	THAU-P1-5_47017	OR578998	THAU-P1-5	THAU	*eptA*	*Vibrio*	MA	20+37°C	857	11,52
**other eptA**	SYLT-P7_16811	OR579015	SYLT-P7	SYLT	*eptA-dgkA*	*Vibrio*	MA	37°C	849	28,11
**other eptA**	EBRO-P4_25721	OR579018	EBRO-P4	EBRO	*dgkA-eptA*	*Vibrio*	MA	20°C	753	11,05
**other eptA**	THAU-P1-5_24140	OR579014	THAU-P1-5	THAU	*dgkA-eptA*	*Vibrio*	MA	20+37°C	226	3,04
**other eptA**	THAU-P3-7_01996	OR579013	THAU-P3-7	THAU	*rstA-rstB-Zipper-eptA*	*Vibrio*	TCBS	20+37°C	192	2,31
**other eptA**	THAU-P1-5_40795	OR579012	THAU-P1-5	THAU	*eptA-arlR*	*Aliivibrio*	MA	20+37°C	177	2,38
**other eptA**	EBRO-P4_14649	OR579020	EBRO-P4	EBRO	*Zipper-eptA*	*Vibrio*	MA	20°C	883	12,96
**other eptA**	THAU-P1-5_36515	OR579011	THAU-P1-5	THAU	*eptA-PGT1-arlR-sasA*	*Photobacterium*	MA	20+37°C	230	3,09
**other eptA**	EBRO-P4_29453	OR579016	EBRO-P4	EBRO	*eptA*	*Aliivibrio*	MA	20°C	953	13,99
other eptA	SYLT-P7_12619	OR579022	SYLT-P7	SYLT	*dgkA-hpap2-eptA*	*Halomonas*	MA	37°C	954	31,58
**other eptA**	EBRO-P6_12023	OR579019	EBRO-P6	EBRO	*dgkA-eptA*	*Vibrio*	TCBS	37°C	225	17,76
**other eptA**	EBRO-P4_29773	OR579017	EBRO-P4	EBRO	*dgkA-eptA-PGT1*	*Vibrio*	MA	20°C	373	5,47
other mcr	EBRO-P4_00710	OR579026	EBRO-P4	EBRO	*mcr*	*Vibrio*	MA	20°C	580	8,51
other mcr	SYLT-P4_10061	OR579023	SYLT-P4	SYLT	*mcr-dgkA*	*Photobacterium*	TCBS	20°C	517	6,74
other mcr	SYLT-P4_06950	OR579024	SYLT-P4	SYLT	*mcr*	*Shewanella*	TCBS	20°C	754	9,83
other mcr	EBRO-P6_04283	OR579027	EBRO-P6	EBRO	*mcr-dgkA*	*Vibrio*	TCBS	37°C	211	16,66
other mcr	THAU-P1-5_15158	OR579021	THAU-P1-5	THAU	*mcr-dgkA*	*Vibrio*	MA	20+37°C	292	3,93
other mcr	EBRO-P8_21207	OR579029	EBRO-P8	EBRO	*mcr-dgkA*	*Pseudoalteromonas*	MA	37°C	154	4,35
other mcr	SYLT-P4_00705	OR579025	SYLT-P4	SYLT	*mcr*	*Shewanella*	TCBS	20°C	386	5,03
other mcr	EBRO-P8_16485	OR579028	EBRO-P8	EBRO	*mcr-dgkA*	*Vibrio*	MA	37°C	721	20,38

Table S3. Amino acid sequence identity between EptA variants

	**VF_A0210**	EBRO-P4_29453|OR579016|eptA-like	THAU-P1-5_40795|OR579012|eptA-like	SYLT-P7_16811|OR579015|eptA-like	THAU-P1-5_36515|OR579011|eptA-like	SYLT-P1_02880|OR579003|eptA-4	EBRO-P4_25590|OR579006|eptA-4	SYLT-P4_02050|OR579001|eptA-4	EBRO-P2_00375|OR579005|eptA-4	SYLT-P1_02695|OR579002|eptA-4	EBRO-P4_30821|OR579010|eptA-4	SYLT-P4_00252|OR579000|eptA-4	EBRO-P4_20292|OR579007|eptA-4	EBRO-P2_05228|OR579004|eptA-4	EBRO-P4_37349|OR579008|eptA-4	SYLT-P4_02614|OR578999|eptA-4	THAU-P3-7_15942|OR578997|eptA-4	EBRO-P4_27855|OR579009|eptA-4	THAU-P1-5_47017|OR578998|eptA-4	EBRO-P2_00219|OR578989|eptA-2	EBRO-P4_18218|OR578990|eptA-2	EBRO-P8_23965|OR578991|eptA-2	EBRO-P2_03786|OR578981|eptA-1	THAU-P1-5_20040|OR578980|eptA-1	EBRO-P4_30301|OR578985|eptA-1	EBRO-P6_09496|OR578982|eptA-1	EBRO-P6_10674|OR578983|eptA-1	EBRO-P8_20143|OR578986|eptA-1	EBRO-P2_02270|OR578984|eptA-1	EBRO-P4_33592|OR578987|eptA-1	EBRO-P8_12622|OR578988|eptA-1	THAU-P3-7_06040|OR578979|eptA-1	EBRO-P6_08759|OR578995|eptA-3	THAU-P1-5_15587|OR578993|eptA-3	THAU-P3-7_04605|OR578992|eptA-3	EBRO-P6_03152|OR578994|eptA-3	EBRO-P8_28758|OR578996|eptA-3	THAU-P1-5_24140|OR579014|eptA-like	THAU-P3-7_01996|OR579013|eptA-like	**VP_RS21300**	**VVMO6_RS15980**	**VCA1102**	EBRO-P4_14649|OR579020|eptA-like	EBRO-P6_12023|OR579019|eptA-like	EBRO-P4_25721|OR579018|eptA-like	EBRO-P4_29773|OR579017|eptA-like
**VF_A0210**	100	99	99	51	51	44	44	44	43	43	43	43	43	43	43	43	43	43	43	44	44	44	44	44	43	43	44	44	44	44	44	44	43	42	42	42	42	41	44	43	43	43	40	43	44	42
EBRO-P4_29453|OR579016|eptA-like	99	100	99	51	51	44	44	44	43	43	43	43	43	43	43	43	43	43	43	45	45	45	44	44	44	44	44	44	44	44	44	44	43	43	43	43	42	41	44	43	43	43	41	44	44	42
THAU-P1-5_40795|OR579012|eptA-like	**99**	99	100	52	51	44	44	44	43	43	43	43	43	43	43	43	43	43	43	45	45	45	44	44	44	44	44	44	44	44	44	44	43	43	43	43	42	41	44	43	43	43	41	44	44	42
SYLT-P7_16811|OR579015|eptA-like	51	51	52	100	54	43	43	43	43	43	43	43	43	43	44	42	43	43	42	45	45	45	44	44	44	44	44	44	45	45	44	44	42	42	42	42	42	41	44	43	44	41	41	41	42	42
THAU-P1-5_36515|OR579011|eptA-like	51	51	51	54	100	43	43	43	43	43	43	43	41	41	41	41	42	42	42	45	45	45	45	44	44	44	44	44	44	44	44	44	42	42	42	43	43	44	44	43	44	42	42	43	43	41
SYLT-P1_02880|OR579003|eptA-4	44	44	44	43	43	100	100	100	94	94	94	94	86	87	86	88	90	89	89	53	53	53	53	53	53	53	53	53	53	53	53	52	53	53	53	53	53	52	54	54	55	54	50	51	50	50
EBRO-P4_25590|OR579006|eptA-4	44	44	44	43	43	100	100	100	94	94	94	94	86	86	86	88	89	89	88	53	53	53	53	53	53	53	53	53	53	53	52	52	52	52	52	53	52	52	54	54	55	54	50	51	50	50
SYLT-P4_02050|OR579001|eptA-4	44	44	44	43	43	100	100	100	94	94	94	94	86	86	86	88	89	89	88	53	53	53	53	53	53	53	53	53	53	53	52	52	52	52	52	53	52	52	54	54	55	54	50	51	50	50
EBRO-P2_00375|OR579005|eptA-4	43	43	43	43	43	94	94	94	100	97	97	97	87	88	87	88	90	90	90	53	53	53	54	54	54	54	54	54	54	54	53	53	53	53	53	54	54	54	55	54	56	**55**	50	51	51	50
SYLT-P1_02695|OR579002|eptA-4	43	43	43	43	43	94	94	94	97	100	99	99	87	88	88	88	90	91	91	54	54	54	54	54	54	54	54	54	54	54	53	53	54	54	54	54	54	54	55	**55**	56	**55**	51	51	51	51
EBRO-P4_30821|OR579010|eptA-4	43	43	43	43	43	94	94	94	97	99	100	100	87	87	87	88	90	91	91	54	54	54	54	54	54	54	54	54	54	54	54	53	54	54	54	54	54	54	55	**55**	**57**	**55**	51	52	51	51
SYLT-P4_00252|OR579000|eptA-4	43	43	43	43	43	94	94	94	97	99	100	100	87	87	87	88	90	91	91	54	54	54	54	54	54	54	54	54	54	54	54	53	54	54	54	54	54	54	55	**55**	**57**	**55**	51	52	51	51
EBRO-P4_20292|OR579007|eptA-4	43	43	43	43	41	86	86	86	87	87	87	87	100	94	94	90	90	91	91	52	52	52	52	52	52	52	53	53	52	52	51	51	52	52	52	52	52	52	53	53	55	55	49	50	49	50
EBRO-P2_05228|OR579004|eptA-4	43	43	43	43	41	87	86	86	88	88	87	87	94	100	99	93	91	92	91	53	53	53	53	53	53	53	54	54	53	53	52	52	52	52	52	52	52	53	53	54	56	55	49	51	49	50
EBRO-P4_37349|OR579008|eptA-4	43	43	43	44	41	86	86	86	87	88	87	87	94	99	100	93	91	91	91	52	52	52	53	53	53	53	53	53	53	53	52	52	52	52	52	52	52	52	53	53	56	55	49	51	49	50
SYLT-P4_02614|OR578999|eptA-4	43	43	43	42	41	88	88	88	88	88	88	88	90	93	93	100	94	93	92	52	52	52	52	52	52	52	53	53	53	53	52	52	54	54	54	54	53	52	53	55	56	55	49	52	49	50
THAU-P3-7_15942|OR578997|eptA-4	43	43	43	43	42	90	89	89	90	90	90	90	90	91	91	94	100	94	93	53	53	53	53	53	53	53	54	54	54	54	54	53	53	53	53	53	52	52	54	54	56	54	49	51	49	49
EBRO-P4_27855|OR579009|eptA-4	43	43	43	43	42	89	89	89	90	91	91	91	91	92	91	93	94	100	96	52	52	52	53	53	53	53	53	53	52	52	53	52	53	53	53	53	53	53	54	54	56	55	50	52	50	50
THAU-P1-5_47017|OR578998|eptA-4	43	43	43	42	42	89	88	88	90	91	91	91	91	91	91	92	93	96	100	52	52	52	52	52	52	52	53	53	52	52	52	52	54	54	54	53	53	52	54	54	55	54	49	52	50	50
EBRO-P2_00219|OR578989|eptA-2	44	45	45	45	45	53	53	53	53	54	54	54	52	53	52	52	53	52	52	100	100	99	87	88	87	87	88	88	87	87	87	87	79	79	78	79	79	76	82	**84**	**73**	**59**	62	65	61	62
EBRO-P4_18218|OR578990|eptA-2	44	45	45	45	45	53	53	53	53	54	54	54	52	53	52	52	53	52	52	100	100	100	87	87	87	87	87	87	86	87	87	86	78	78	78	79	78	76	82	83	73	59	62	65	61	62
EBRO-P8_23965|OR578991|eptA-2	44	45	45	45	45	53	53	53	53	54	54	54	52	53	52	52	53	52	52	99	100	100	87	87	87	87	87	87	86	87	87	87	78	78	78	79	78	76	82	83	73	59	62	65	61	62
EBRO-P2_03786|OR578981|eptA-1	44	44	44	44	45	53	53	53	54	54	54	54	52	53	53	52	53	53	52	87	87	87	100	100	99	99	92	92	91	91	92	92	77	77	77	77	77	76	81	80	70	58	61	64	62	62
THAU-P1-5_20040|OR578980|eptA-1	44	44	44	44	44	53	53	53	54	54	54	54	52	53	53	52	53	53	52	88	87	87	100	100	100	100	92	92	91	91	92	92	77	77	77	77	77	76	81	80	71	58	62	64	62	62
EBRO-P4_30301|OR578985|eptA-1	43	44	44	44	44	53	53	53	54	54	54	54	52	53	53	52	53	53	52	87	87	87	99	100	100	100	92	92	91	91	92	92	77	77	77	77	76	76	81	80	70	58	61	63	62	61
EBRO-P6_09496|OR578982|eptA-1	43	44	44	44	44	53	53	53	54	54	54	54	52	53	53	52	53	53	52	87	87	87	99	100	100	100	92	92	91	91	92	92	77	77	77	77	76	76	81	80	70	58	61	63	62	61
EBRO-P6_10674|OR578983|eptA-1	44	44	44	44	44	53	53	53	54	54	54	54	53	54	53	53	54	53	53	88	87	87	92	92	92	92	100	99	93	93	94	94	78	78	78	79	79	77	81	**81**	**71**	**58**	61	63	63	61
EBRO-P8_20143|OR578986|eptA-1	44	44	44	44	44	53	53	53	54	54	54	54	53	54	53	53	54	53	53	88	87	87	92	92	92	92	99	100	93	93	95	94	78	78	78	79	78	77	81	81	71	58	61	63	62	61
EBRO-P2_02270|OR578984|eptA-1	44	44	44	45	44	53	53	53	54	54	54	54	52	53	53	53	54	52	52	87	86	86	91	91	91	91	93	93	100	100	95	95	77	77	77	77	77	77	81	81	69	58	61	63	61	60
EBRO-P4_33592|OR578987|eptA-1	44	44	44	45	44	53	53	53	54	54	54	54	52	53	53	53	54	52	52	87	87	87	91	91	91	91	93	93	100	100	95	95	77	77	77	77	77	77	81	**81**	69	58	61	63	61	60
EBRO-P8_12622|OR578988|eptA-1	44	44	44	44	44	53	52	52	53	53	54	54	51	52	52	52	54	53	52	87	87	87	92	92	92	92	94	95	95	95	100	99	77	77	77	77	77	78	81	80	70	58	61	63	61	61
THAU-P3-7_06040|OR578979|eptA-1	44	44	44	44	44	52	52	52	53	53	53	53	51	52	52	52	53	52	52	87	86	87	92	92	92	92	94	94	95	95	99	100	77	77	77	78	77	77	80	80	70	57	61	63	61	60
EBRO-P6_08759|OR578995|eptA-3	43	43	43	42	42	53	52	52	53	54	54	54	52	52	52	54	53	53	54	79	78	78	77	77	77	77	78	78	77	77	77	77	100	99	99	92	91	77	77	83	70	57	60	63	59	63
THAU-P1-5_15587|OR578993|eptA-3	42	43	43	42	42	53	52	52	53	54	54	54	52	52	52	54	53	53	54	79	78	78	77	77	77	77	78	78	77	77	77	77	99	100	100	92	91	77	77	83	70	57	60	63	59	63
THAU-P3-7_04605|OR578992|eptA-3	42	43	43	42	42	53	52	52	53	54	54	54	52	52	52	54	53	53	54	78	78	78	77	77	77	77	78	78	77	77	77	77	99	100	100	92	91	77	77	83	70	57	60	62	59	63
EBRO-P6_03152|OR578994|eptA-3	42	43	43	42	43	53	53	53	54	54	54	54	52	52	52	54	53	53	53	79	79	79	77	77	77	77	79	79	77	77	77	78	92	92	92	100	99	78	78	**84**	**70**	**58**	61	63	58	62
EBRO-P8_28758|OR578996|eptA-3	42	42	42	42	43	53	52	52	54	54	54	54	52	52	52	53	52	53	53	79	78	78	77	77	76	76	79	78	77	77	77	77	91	91	91	99	100	78	78	83	70	58	60	63	58	62
THAU-P1-5_24140|OR579014|eptA-like	41	41	41	41	44	52	52	52	54	54	54	54	52	53	52	52	52	53	52	76	76	76	76	76	76	76	77	77	77	77	78	77	77	77	77	78	78	100	76	79	69	57	60	61	60	61
THAU-P3-7_01996|OR579013|eptA-like	44	44	44	44	44	54	54	54	55	55	55	55	53	53	53	53	54	54	54	82	82	82	81	81	81	81	81	81	81	81	81	80	77	77	77	78	78	76	100	80	69	57	61	64	61	61
**VP_RS21300**	43	43	43	43	43	54	54	54	54	55	55	55	53	54	53	55	54	54	54	84	83	83	80	80	80	80	81	81	81	81	80	80	83	83	83	84	83	79	80	100	70	58	61	64	61	62
**VVMO6_RS15980**	43	43	43	44	44	55	55	55	56	56	57	57	55	56	56	56	56	56	55	73	73	73	70	71	70	70	71	71	69	69	70	70	70	70	70	70	70	69	69	70	100	60	62	63	62	66
**VCA1102**	43	43	43	41	42	54	54	54	55	55	55	55	55	55	55	55	54	55	54	59	59	59	58	58	58	58	58	58	58	58	58	57	57	57	57	58	58	57	57	58	60	100	54	53	55	57
EBRO-P4_14649|OR579020|eptA-like	40	41	41	41	42	50	50	50	50	51	51	51	49	49	49	49	49	50	49	62	62	62	61	62	61	61	61	61	61	61	61	61	60	60	60	61	60	60	61	61	62	54	100	64	57	56
EBRO-P6_12023|OR579019|eptA-like	43	44	44	41	43	51	51	51	51	51	52	52	50	51	51	52	51	52	52	65	65	65	64	64	63	63	63	63	63	63	63	63	63	63	62	63	63	61	64	64	63	53	64	100	55	57
EBRO-P4_25721|OR579018|eptA-like	44	44	44	42	43	50	50	50	51	51	51	51	49	49	49	49	49	50	50	61	61	61	62	62	62	62	63	62	61	61	61	61	59	59	59	58	58	60	61	61	62	55	57	55	100	58
EBRO-P4_29773|OR579017|eptA-like	42	42	42	42	41	50	50	50	50	51	51	51	50	50	50	50	49	50	50	62	62	62	62	62	61	61	61	61	60	60	61	60	63	63	63	62	62	61	61	62	66	57	56	57	58	100

**% identitty**	40	50	60	70	80	90	##

Table S4. Strains and Plasmids

Bacterial strains	Superclade	Isolation Source	Temp	Description	Reference	Accession Number	Conting number	size (Mb)	CDSs	Coverage
Vibrio harveyi Th15_Z_F11	Harveyi	Water column, Thau lagoon, France	20ºC	eptA-1; [Col-R]	(Oyanedel et al., 2023)	ERS9919775	53	5,7	5395	
Vibrio harveyi Th15_F5_F11	Harveyi	Water column, Thau lagoon, France	20ºC	eptA-1; [Col-R]	(Oyanedel et al., 2023)	ERS9919777	106	5,9	5524	
Vibrio jasicida Th15_F5_H11	Harveyi	Water column, Thau lagoon, France	20ºC	eptA-1; [Col-R]	(Oyanedel et al., 2023)	ERS9919787	52	6,1	5712	
Vibrio owensii Th15_Z_G08	Harveyi	Water column, Thau lagoon, France	20ºC	eptA-1; [Col-R]	(Oyanedel et al., 2023)	ERS9919782	98	6,0	5566	
Vibrio rotiferianus Th15_O_H08	Harveyi	Oyster, Thau lagoon, France	20ºC	eptA-2; [Col-R]	(Oyanedel et al., 2023)	ERS9919779	81	5,2	4835	
Vibrio rotiferianus Th15_O_G05	Harveyi	Oyster, Thau lagoon, France	20ºC	eptA-2; [Col-R]	(Oyanedel et al., 2023)	ERS9919778	85	5,3	4956	
Vibrio splendidus 7T7_2	Harveyi	Oyster, Bay of Brest, France	20ºC	eptA-4; [Col-S]	(Bruto et al., 2016)		297	5,9	5594	
Vibrio crassostreae J2-4	Splendidus	Oyster, Bay of Brest, France	20ºC	eptA-4; [Col-S]	(Lemire et al., 2015)	PRJEB5888	84	5,7	5285	
Vibrio crassostreae J2-14	Splendidus	Oyster, Bay of Brest, France	20ºC	eptA-4; [Col-S]	(Lemire et al., 2015)	PRJEB5892	65	5,7	5244	
Vibrio crassostreae J5-15	Splendidus	Oyster, Bay of Brest, France	20ºC	eptA-4; [Col-S]	(Lemire et al., 2015)	PRJEB5879	92	5,7	5345	
Vibrio sp. TH21_20_OG1	unknown	Oyster, Thau lagoon, France	20ºC	eptA-, mcr-; [Col-R]	this study	ERR12116512	56	5,4	4945	78X
Vibrio sp. TH21_20_OH4	unknown	Oyster, Thau lagoon, France	20ºC	eptA-, mcr-; [Col-R]	this study	ERR12116510	76	5,4	4903	34X
Vibrio sp. TH21_20A_OC7	unknown	Oyster, Thau lagoon, France	20ºC	eptA-, mcr-; [Col-R]	this study	ERR12116511	76	5,4	4790	68X

Vibrio sp. TH21_20A_OB7	Harveyi	Oyster, Thau lagoon, France	20ºC	eptA-1; [Col-R]	this study	ERR12116517	177	6	5667	8X
Vibrio jasicida TH21_20A_OE8	Harveyi	Oyster, Thau lagoon, France	20ºC	eptA-1; [Col-R]	this study	ERR12116518	215	6,3	5824	33X
Vibrio owensii TH21_37_OE7	Harveyi	Oyster, Thau lagoon, France	37ºC	eptA-1; [Col-R]	this study	ERR12116513	71	5,9	5438	47X
Vibrio alginolyticus TH21_37_OE9	Harveyi	Oyster, Thau lagoon, France	37ºC	eptA-3; [Col-R]	this study	ERR12116514	68	5,3	4893	54X
Vibrio alginolyticus TH21_37A_OE10	Harveyi	Oyster, Thau lagoon, France	37ºC	eptA-3; [Col-R]	this study	ERR12116515	75	5,1	4690	53X
Vibrio alginolyticus TH21_37A_OE12	Harveyi	Oyster, Thau lagoon, France	37ºC	eptA-3; [Col-R]	this study	ERR12116516	88	5,3	4877	25X
Vibrio harveyi TH15_F5_F11 Δ*rstA*	Harveyi	na	20ºC	*DrstA*	this study					
Vibrio harveyi TH15_F5_F11 Δ*rstA* pMRB-P_{LAC}-rstA	Harveyi	na	20ºC	Δ*rstA, rstA* under plac promoter in pMRB plasmid	this study					
*Vibrio harvey*i TH15_F5_F11 Δ*rstA* pMRB-P_{LAC}-gfp	Harveyi	na	20ºC	Δ*rstA, gfp* under plac promoter in pMRB plasmid	this study					
E. coli P3813		na	37ºC	*lacIQ, thi1, supE44, endA1, recA1, hsdR17, gyrA462, zei298::Tn10,* D*thyA::(erm-pir116)* [Tc^R Erm^R]	(Le Roux et al., 2007)					
E. coli b3914		na	37ºC	(F^-) RP4-2-Tc::Mu DdapA ::*(erm-pir116), gyrA462,*	(Le Roux et al., 2007)					

				zei298::Tn10 [Km^R Em^R Tc^R]	
E. coli TOP10		na	37ºC	TOP10 Chemically Competent E. coli	Invitrogen
Vibrio parahaemolyticus IFVp22	Harveyi	Mussel, English channel, France	37ºC	eptA; [Col-R]	(Sorée M. et al., 2024)
Vibrio vulnificus CECT4999	Vulnificus	Eel, Spain	37ºC	eptA; [Col-R]	(Murciano C. et al., 2017)

**Plasmids**	**Description**	**Reference**
pSW7848T	*oriV*$_{R6K}$; *oriT*$_{RP4}$; *araC*-P_{BAD}*ccdB;* [CmR]	(Val et al., 2012)
pSW7848 Δ*rstA*	pSW7848T :: ΔrstA	this study
pMRB-P_{LAC}-*gfp*	*oriVR6Kg ; oriTRP4; oriVpB1067;* P_{LAC}-*gfp* [CmR]	(Le Roux et al., 2011)
pMRB-P_{LAC}-rstA	*oriVR6Kg ; oriTRP4; oriVpB1067*P_{LAC}-rstA [CmR]	this study
pBAD-TOPO	pBAD TOPO® TA Expression Kit (Catalog nos. K4300-01, K4300-40)	Invitrogen
pBAD-TOPO-*dgkA-eptA-1*	*dgkA-eptA-1* under pBAD promoter in pBAD TOPO® TA	this study
pBAD-TOPO-*eptA-1*	*eptA-1* under pBAD promoter in pBAD TOPO® TA	this study
pBAD-TOPO-*dgkA*	*dgkA* under pBAD promoter in pBAD TOPO® TA	this study
pBAD-TOPO-*eptA-4*	*eptA-4* under pBAD promoter in pBAD TOPO® TA	this study

Table S5. List of primers used in this study

Name	Sequence 5'-3'	Description	DNA matrix
240423-1	GTATCGATAAGCTTGATATCGAATTCCCTTCTCTAATGCCCTTACG	*rst*A gene deletion-1	*Vibrio harveyi* Th15_F5_F11
240423-2	GACGATCCTAAACTGCAAGCCCCTGATGCTTGGAATGCGTAATG	*rst*A gene deletion-2	*Vibrio harveyi* Th15_F5_F11
240423-3	CATTACGCATTCCAAGCATCAGGGGCTTGCAGTTTAGGATCGTC	*rst*A gene deletion-3	*Vibrio harveyi* Th15_F5_F11
240423-4	CCCCCGGGCTGCAGGAATTCTGTTTCGTCTGTTCTGGTGC	*rst*A gene deletion-4	*Vibrio harveyi* Th15_F5_F11
120523-1	GTGAGCGGATAACAAAGGAAGGGCCCATGAATGTACAAGACACTAAGC	*rst*A complementation (forward)	*Vibrio harveyi* Th15_F5_F11
120523-2	CGACGCGTCTGCAGCTCGAGTTACGCATTCCAAGCATCAG	*rst*A complementation (reverse)	*Vibrio harveyi* Th15_F5_F11
240423-10	AGTGCACCTGCGCCAGCACC	PCR *rst*A (forward)	*Vibrio harveyi* Th15_F5_F11
240423-11	AGGCATCGCAACTAAACAGC	PCR *rst*A (reverse)	*Vibrio harveyi* Th15_F5_F11
V1-dgkA-eptA-F	CACCAGGAGATAATAATTGAAACCAGGTAAAACAGG	*dgk*A cloning (forward)	*Vibrio owensii* Th15_Z_G08
V1-dgkA-R	CTAAAGTAAAATCGACGCCCAAACAAATAGGG	*dgk*A cloning (reverse)	*Vibrio owensii* Th15_Z_G08
V1-eptA-F	CACCAGGAGATAATAAATGAAGACAATTGAACTCCC	*ept*A-1 cloning (forward)	*Vibrio owensii* Th15_Z_G08
V1-dgkA-eptA-R	TTAGCTTTGACGACAAGTCTTAAAGATATCTAAAGCG	*ept*A-1 cloning (reverse)	*Vibrio owensii* Th15_Z_G08
V4-eptA-F	CACCAGGAGATAATAAATGAATCTACCACGCTTAAAC	*ept*A-4 cloning (forward)	*Vibrio splendidus* 7T7-2
V4-eptA-R	CTACCGACACGCAGCAAAAATGTC	*ept*A-4 cloning (reverse)	*Vibrio splendidus* 7T7-3
dgkA_32F	GGCTCAGCAGTATAGGTCCC	*dgk*A RT-qPCR (forward)	*Vibrio harveyi* Th15_F5_F11
dgkA_94R	CGTCCAATATCCGGCGAATG	*dgk*A RT-qPCR (reverse)	*Vibrio harveyi* Th15_F5_F11
eptA_175F	TTTGCAGCGCTGAACTTCTT	*ept*A-1 RT-qPCR (forward)	*Vibrio harveyi* Th15_F5_F11
eptA_296R	AGTGTGCCGTAGTTGTAGCT	*ept*A-1 RT-qPCR (reverse)	*Vibrio harveyi* Th15_F5_F11
VH_2913_640F	AAGAAGCACGCAATCATCGC	VH_2913 RT-qPCR (forward)	*Vibrio harveyi* Th15_F5_F11
VH_2913_750R	GTGACCAAGAACCGTTGCAC	VH_2913 RT-qPCR (reverse)	*Vibrio harveyi* Th15_F5_F11
VH_0852_53F	CCCATGCAGCGATTGAAGTG	VH_0852 RT-qPCR (forward)	*Vibrio harveyi* Th15_F5_F11
VH_0852_185R	TGAGCCAATTCTGCATTCGA	VH_0852 RT-qPCR (reverse)	*Vibrio harveyi* Th15_F5_F11
